# Recent Strategies and Advances in Hydrogel-Based Delivery Platforms for Bone Regeneration

**DOI:** 10.1007/s40820-024-01557-4

**Published:** 2024-11-27

**Authors:** Xiao Wang, Jia Zeng, Donglin Gan, Kun Ling, Mingfang He, Jianshu Li, Yongping Lu

**Affiliations:** 1 Scientific and Technological Innovation Center for Biomedical Materials and Clinical Research, Guangyuan Key Laboratory of Multifunctional Medical Hydrogel, Guangyuan Central Hospital, Guangyuan, 628000 People’s Republic of China; 2 Jiangsu Collaborative Innovation Center of Biomedical Functional Materials, Jiangsu Key Laboratory of Bio-Functional Materials, School of Chemistry and Materials Science, Nanjing Normal University, Nanjing, 210023 People’s Republic of China; 3 College of Polymer Science and Engineering, State Key Laboratory of Polymer Materials Engineering, Sichuan University, Chengdu, 610065 People’s Republic of China

**Keywords:** Hydrogel, Bone regeneration, Bioactive molecules, Drug delivery, Nano-/microscale carriers

## Abstract

Bioactive molecules have shown great promise for effectively regulating various bone formation processes, rendering them attractive therapeutics for bone regeneration. However, the widespread application of bioactive molecules is limited by their low accumulation and short half-lives in vivo. Hydrogels have emerged as ideal carriers to address these challenges, offering the potential to prolong retention times at lesion sites, extend half-lives in vivo and mitigate side effects, avoid burst release, and promote adsorption under physiological conditions. This review systematically summarizes the recent advances in the development of bioactive molecule-loaded hydrogels for bone regeneration, encompassing applications in cranial defect repair, femoral defect repair, periodontal bone regeneration, and bone regeneration with underlying diseases. Additionally, this review discusses the current strategies aimed at improving the release profiles of bioactive molecules through stimuli-responsive delivery, carrier-assisted delivery, and sequential delivery. Finally, this review elucidates the existing challenges and future directions of hydrogel encapsulated bioactive molecules in the field of bone regeneration.

## Highlights

- Recent advances in the combined delivery platform that integrate nano-/microscale carriers and with 3D hydrogel network for bone regeneration are summarized.
- The strategies for bioactive molecules delivery involving nanoparticles, nanosheets, and microspheres, along with extra stimuli such as near-infrared light, temperature changes, ultrasonication, and inflammatory conditions, are introduced.
- The prospects and challenges for the clinical translation and the development of nano-/microscale incorporated hydrogel-based delivery platform are discussed.

## Graphical abstract

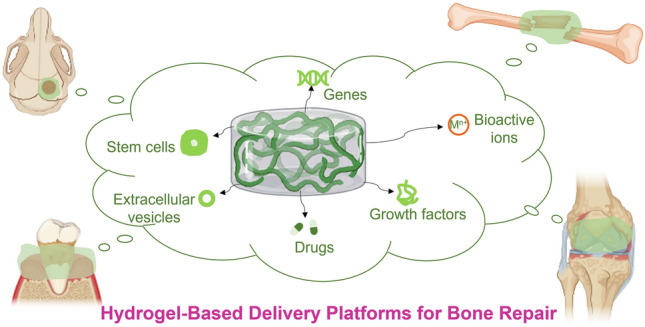

## Introduction

Bone tissue is a vital component of the human body, comprising one of the fundamental organ systems that provides essential support for movement, and playing a pivotal role in facilitating physical activity. Additionally, bone tissue can safeguard the vital organs and regulate the cellular metabolism. Consequently, maintaining the health of bone tissue is imperative for fostering social participation and is a critical determinant of an individual’s quality of life [1]. However, approximately 50% of adults, and particularly the aged population, experience bone injuries or defects [2]. These issues are usually induced by trauma, diseases, and other factors [3, 4]. The gold standard treatments for bone repair predominantly involve autografts, allografts, and internal fixation [5, 6]. Although these approaches have gained widespread acceptance, certain drawbacks are evident, including the limited availability of donor tissues, risk of infection, potential immunogenicity, and other associated concerns [7–9].

Bone tissue exhibits inherently dynamic and vascularized characteristics, thereby rendering it highly regenerative. Indeed, controlling the osteoblast function has been demonstrated to significantly enhance new bone formation and increase bone mass [10]. Therefore, various bioactive molecules, including drugs [11, 12], growth factors (GFs) [13, 14], stem cells [15], extracellular vesicles (EVs) [16, 17], and bioactive ions [18, 19], have been applied to bone regeneration in various pathological conditions. These molecules act directly on the injured bone tissue and demonstrate satisfactory therapeutic efficacies. However, several challenges remain unaddressed. For instance, a low in vivo stability and poor retention capability at the lesion site may require the high-dose administration of bioactive molecules, causing an increased toxicity toward normal tissues, and the potential for developing multidrug resistance. Generally, bone regeneration is a time-consuming process, rendering it crucial to construct a suitable platform for delivering bioactive molecules to injured bone sites and achieving a sustained release. In recent years, several nanomaterial-based carriers, including nanoparticles (NPs) [20, 21], graphene oxide (GO) /black phosphorus (BP) nanosheets (NSs) [22, 23], metal–organic frameworks (MOFs) [24, 25], and nanomicelles [26, 27], have been proposed as delivery systems for bioactive molecules. However, the direct administration of these carriers is essential for further discussion because of their elusive in vivo distributions, low plasma stability, and high does-induced potential toxicities toward normal tissues. The usage of nanomaterial-based carriers for the delivery of bioactive molecules is still a developing field and requires further research.

The generation of novel biomaterial-mediated therapeutics offers potential to effectively enhance the sequential and spatial delivery of bioactive molecules or cell therapy to injured bone sites. Hydrogels are particularly appealing because of their tissue-like structures, high water contents within network, good biocompatibility, controllably physicochemical properties, and their ability to serve as carriers for the release of therapeutic molecules. Hydrogels can reduce the potential toxicities and side effects generally associated with drug-directed delivery, while also extending the local drug retention rate through their slow but gradual biodegradability and swelling properties in vivo. Furthermore, hydrogels exhibit multifunctional stimuli-responsive properties that can be triggered by various factors, such as pH [28], near-infrared (NIR) irradiation [11, 29], reactive oxygen species (ROS) [30, 31], and ultrasonic (US) stimulation [32, 33] at the lesion site. This ensures the controllable release of bioactive molecules from the hydrogel network, avoiding the unnecessary waste of therapeutic agents. Therefore, hydrogel-based spatial/sequential delivery is a promising strategy for bone repair when aligned with the natural bone healing process. These advantages render hydrogels ideal delivery platforms for loading various therapeutic molecules either proper physical encapsulation or chemical conjugation.

**Fig. 1:**
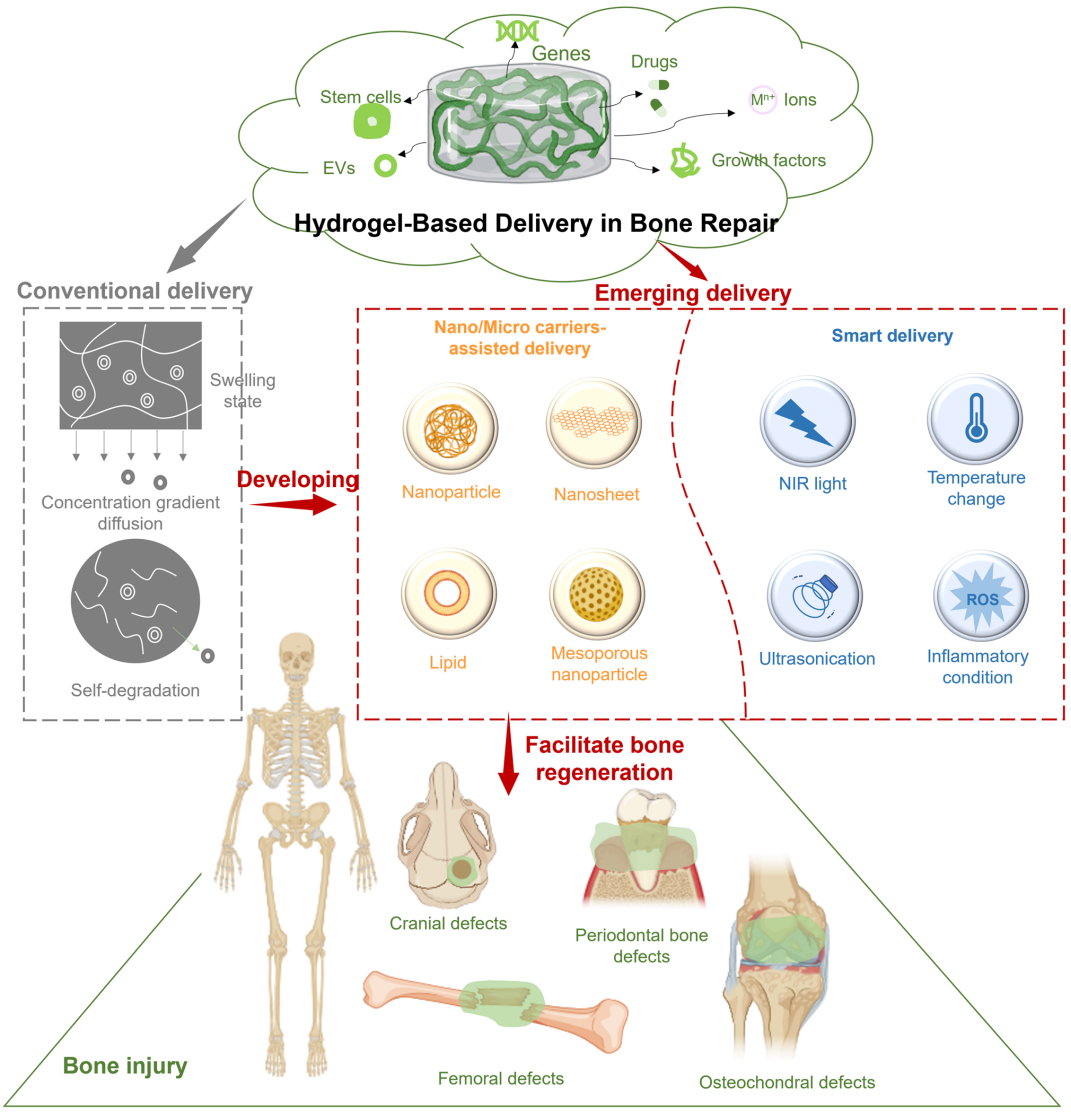
Examples of hydrogel-based delivery platforms for bone regeneration. Common bone injuries are also shown, including cranial defects, femoral defects, periodontal bone defects, and osteochondral defects. To repair bone defects, conventional hydrogels deliver bioactive molecules via their inherent biodegradability or swelling properties. Emerging deliver systems employ hydrogels as matrices for drug delivery with the assistance of nano-/microcarriers, additional stimuli, or stratified structures. (Created with MedPeer (medpeer.cn))

To date, various exciting studies have reported the development of hydrogel-based delivery systems for bioactive molecules in bone regeneration (Fig. 1). Although some reviews have summarized hydrogel-based delivery for biomedical applications, the majority have focused on a single class of therapeutic delivery for various purposes. Therefore, this review presents a systematic overview of the strategies reported for developing hydrogel platforms, in addition to recent advances in bone reconstruction, and details regarding the bio-functions of various bioactive molecules during osteogenesis. Special attention has been given to hydrogel delivery systems reported in the last five years, emphasizing multifunctional US-, pH-, ROS-, and NIR-responsive materials, as well as sustained release for long-term therapy. This review highlights the importance of the release behaviors and pharmacokinetics of bioactive molecules in achieving an effective therapy. The opportunities and current challenges in the future development of hydrogel delivery platforms for bone regeneration are also explored.

## Bioactive Molecules for Bone Regeneration

After injury, the bone healing process can be divided into three stages, namely the inflammatory process, bone formation, and the remodeling process [34]. It is therefore important to develop a platform that controls the release of bioactive molecules at an appropriate time to achieve an optimal efficacy. Bioactive molecules, primarily drugs, bioactive ions, GFs, stem cell, EVs, and genes are frequently integrated into hydrogels to increase their stabilities and maintain an optimal biological efficiency at the defect site, thereby enhancing new bone formation as quickly as possible.

### Growth Factors

GFs with inherent bone-inducing properties are classified as bioactive molecules and have drawn considerable attention in the field of bone regeneration. These GFs (e.g., bone morphogenic protein (BMP-2), vascular endothelial growth factor (VEGF), and stromal derived factor-1*α* (SDF-1*α*)) bind to the membrane of various cells to exert their bioactivities, including inflammatory suppression, angiogenesis, and osteogenesis. Thus, GFs have emerged as promising therapeutic drugs candidates. The most commonly used GF for bone regeneration is BMP-2, which has been approved by the United States Food and Drug Administration (US FDA). BMP-2 is a key factor to accelerate bone regeneration and shows strong osteogenic ability. BMP-2 can not only enhance osteogenic differentiation, strengthen alkaline phosphatase (ALP) activity, and facilitate mineralization of bone matrix, but also control the mammalian growth and development during bone differentiation process [35]. In addition, BMP-2 also improved the surface regularity, tissue integration of new cartilage via upregulating collagen II (COL II) and aggrecan level around the impaired cartilage [36]. However, its applications are limited by a short half-life of 7 min and low retention rates at the bone defect sites. Although supraphysiological doses of BMP-2 have been clinically used to accelerate bone formation in fractures and large bone defects, the abnormal bone formation caused by BMP-2 burst release from traditional collagen scaffolds results in serious complications [37]. Consequently, the development of a sustained delivery system for GFs such as BMP-2 is necessary. It is well known that the bone is a highly vascularized tissue that requires supply of sufficient nutrients and mineralizing components, which can lead to the simultaneous recruitment of mesenchymal stem cells (MSCs) to the lesion sites. To enhance bone regeneration, the VEGF is used to as an angiogenic factor to induce vascularization [38]. Similarly, SDF-1*α* (also known as CXCL12) has been reported to recruit bone mesenchymal stem cells (BMSCs) to bone defects via its chemotactic effects [39–42]. In addition, platelet-rich plasma (PRP), belongs to an autologous and economical GF containing platelet-derived growth factor (PDGF), transforming growth factor-*β* (TGF-*β*), and SDF-1*α*, have been developed to accelerate the migration, proliferation, and differentiation of BMSCs toward osteogenesis and chondrogenesis [43, 44]. Furthermore, PRP is also able to upregulate the expression levels of M2-related genes (e.g., Arg-1 and CD206) [45]. However, the application of GFs is limited in practice due to their poor stability, high cost, and non-physiological delivery [46]. Consequently, there is an urgent need to fabricate delivery platforms to achieve sustained GFs release for bone regeneration. The detailed bioactivities of the latest GFs applied in bone repair are listed in Table 1.

**Table 1:** Representative GFs applied in bone regeneration

Growth factor	Main bioactivity	Bone repair	References
SDF-1	Trigger the Wnt/*β*-catenin signaling pathway	Periodontal bone repair	[13]
	Recruit BMSCs		[47]
Insulin-like growth factor-1 (IGF-1)	Induce osteogenic differentiation of MSCs by activating mammalian target of rapamycin (mTOR)	Age-related osteoporosis	[48]
BMP-2	Promote MSCs differentiation toward osteoblasts	Craniofacial defects	[14]
		Periodontal bone repair	[47]
		Cranial defect repair	[49]
		Radial bone defects repair	[50]
	Enhance mineralized tissue formation	Cranial defect repair	[51]
Platelet-rich fibrin (PRF)	Promote osteoblast differentiation via the Yes-associated protein pathway	Cranial defect repair	[52]
Bone morphogenetic proteins-4 (BMP-4)	Enhance M2 macrophages polarization to accelerate new bone formation	Cranial defect repair in diabetic rats	[23]
Recombinant human BMP-9 (rhBMP9)	Promote osteogenesis by regulating the phosphorylation of Smad1/5/8/	Mandibular and cranial defect repair	[27]
VEGF	Induce vascular growth at the early stage of angiogenesis to support bone healing	Cranial defect repair	[53]
		Osteonecrosis therapy	[54]
PDGF-BB	Recruit smooth muscle cells to construct a mature vascular network to support bone healing process	Cranial defect repair	[53]
Cementum protein 1 (CEMP1)	Promote cementogenic differentiation	Periodontal defect repair	[55]
PRP-derived growth factors	Promote alveolar bone repair		
BMP-7	Promote BMSCs osteogenic and chondrogenic differentiation	Osteochondral defect repair	[56]
	Facilitate osteogenic differentiation of stem cells by strengthening Smad, p38 MARK, and ERK pathway	Critical-sized cranial defect repair	[57]
	Enhance cartilage repair via upregulating the SRY-type high-mobility group box 9 (SOX-9), COL II, and aggrecan expression level	Cartilage repair	[58]
Phenamil (PM)	Promote osteogenesis via BMP-Smad signaling pathway	Chronic bone infection therapy (Osteomyelitis)	[59]
Fibroblast growth factor 2 (FGF-2)	Enhance bone regeneration via FGFR1 activation and promoting osteogenic differentiation	Critical-sized femoral defect repair	[60]
	Facilitate cartilage repair via activating the FGF/FGFR signaling pathway and participating in various biological activities, such as cell differentiation, immune regulation	Cartilage repair	[61]

### Drugs

Therapeutic drug delivery is a relatively straightforward strategy for effectively treating injured bone tissues via direct oral administration or intravenous injection. In addition to general bone damage, bone loss can also be caused by underlying diseases related to the bone tissue, such as bone tumors, periodontitis, osteomyelitis, and osteoporosis. Therefore, drugs must be developed for the treatment of bone defects triggered by such diseases. To date, various drugs (such as parathyroid hormone (PTH), alendronate sodium (ADA), and sitagliptin) have been approved for clinical use by the US FDA. Clinically, PTH is the first choice in the clinical treatment of osteoporosis, and this achieved through systematic intermittent administration [62–64]. As a crucial regulator of calcium hemostasis [65], PTH can upregulate the levels of serum calcium by enhancing calcium reabsorption in the kidney, and by promoting osteoclast differentiation and proliferation to induce osteoclastic bone resorption and calcium delivery from bone tissues [66]. Many studies have demonstrated that PTH can repair bone defects [67] and accelerate fracture healing [68]. However, local bone defect recovery is difficult to achieve using PTH due to low drug concentrations at the lesion sites, and the side effects caused by the injection of high does. Thus, the controlled release of PTH would play a key role in the successful repair of bone defects. Alternatively, ADA is a promising bisphosphonate for the treatment of skeletal disorders, osteoporosis, and osteolysis. ADA can facilitate MSCs differentiation toward osteogenesis via upregulating the expression of some typical osteogenic markers, such as ALP, Runt-related transcription factor 2 (RUNX2), COL 1, and osteocalcin (OCN) [69]. Then, ADA can suppress osteoclast formation by downregulating the expression of osteoclast-specific gene marker cathepsin K (CTSK). In addition, ADA can also bind to bone mineral and then be absorbed by the mature osteoclasts in the human body, finally triggering osteoclast apoptosis and decreasing bone loss [70]. The oral administration of ADA has been demonstrated to trigger gastrointestinal ulceration and jaw osteonecrosis [71, 72]. As another example, deferoxamine (DFO), a hypoxia-mimetic drug, has been employed to repair damaged angiogenesis systems, which is essential in the context of bone regeneration. DFO is capable of upregulating the signal transduction of hypoxia-inducible factor-1*α* (HIF-1*α*) to mimic the hypoxia microenvironment and improve the expression levels of angiogenic factor VEGF [73, 74]. However, the short half-life and low concentration of DFO achieved at the bone defect sites limit its direct administration in the clinical practice. In patients with bone loss and diabetes, sitagliptin is commonly used to regulate the polarization of M1/M2 macrophages and to promote both angiogenesis and osteogenesis [75]. However, the oral administration of sitagliptin induces a series of adverse side effects, including gastrointestinal and hepatic damage [76]. These examples therefore demonstrate that a controlled local delivery system must be established to prevent drug inactivation and to avoid initial burst release. Table 2 summarizes the latest therapeutic drugs reported for bone regeneration.

**Table 2:** Representative therapeutic drugs that have been applied in bone regeneration

Drug	Main bioactivity	Bone repair	References
PTH-related protein (PTHrP-2)	Promote angiogenesis and maintain the osteoclast/osteoblast balance	Femoral defect repair	[11]
Dimethyloxalylglycine (DMOG)	Trigger angiogenic factors expression	Cranial defect repair	[77]
DFO	Promote osteogenesis and angiogenesis	Femoral defect repair	[78]
PTH	Facilitate osteogenesis via the cAMP/PKA/CREB signaling pathway	Cranial defect repair in osteoporotic rats	[12]
	Regulate the osteoclast/osteoblast balance	Cranial defect repair in osteoporotic rats	[29]
		Cranial defect repair	[79]
ADA	Promote the proliferation and differentiation of BMSCs	Cranial defect repair	[80]
	Inhibit the resorptive activity of mature osteoclasts to induce osteoclast apoptosis	Femoral defect repair in osteoporotic rats	[81]
Dexamethasone (Dex)	Suppress apoptosis and promote osteogenic differentiation of BMSCs	Cranial defect repair	[82]
	Promote stem cell differentiation	Osteoporotic bone repair	[83]
		Femoral defect repair	[84]
Simvastatin (SIM)	Induce osteogenesis by depleting cholesterol to enhance pluripotent precursor-MSCs differentiation to osteoblast	Cranial defects repair in hyperlipidemic rats	[24]
Kaempferol	Inhibit osteoclastogenesis and bone resorption through NF-κB	Osteoporotic fracture repair	[20]
Calcitonin gene related peptide (CGRP)	Promote the osteogenic differentiation of BMSCs	Cranial defect repair at a critical size	[85]
Icaritin	Facilitate endogenous cells recruitment to the bone defects and promote new bone formation	Steroid-associated osteonecrosis (SAON) of the femoral head	[86]
Melatonin (Mel)	Promote osteogenic and cementogenic differentiation by decreasing high endoplasmic reticulum stress and the unfolded protein response	Periodontal bone repair in periodontitis rats	[87]
Atsttrin	Inhibit inflammation; promote BMSCs proliferation, and endochondral bone differentiation	Diabetic fracture repair	[88]
Sitagliptin	Recruit M2 macrophages and promote osteointegration	Femoral defect repair in diabetic rats	[75]
Resveratrol (Res)	Regulate the bone immune system by diminishing the inflammatory response to accelerate bone repair	Fracture healing	[89]
		Osteoporotic bone defect repair	[83]
Metformin (Met)	Promote COL I formation, ALP activity, and BMP-2 secretion; Relieve inflammation	Periodontal bone repair in diabetic rats	[90]
Semaphorin 3A (Sema3A)	Relieve inflammation, inhibit osteoclasts differentiation and promote osteoblasts differentiation	Periodontal bone repair in periodontitis	[91]
Propranolol (PRN)	Inhibit the binding process between catecholamine and the *β*-adrenergic receptor; enhance neuropeptides secretion (neuromodulatory microenvironment)	Cranial defect repair at a critical size	[85]
Quercetin	Reduce oxidative stress and promote the osteogenesis of orofacial MSCs via molecular mechanisms to mediate the change of m6A in Per1	Periodontal bone repair in periodontitis	[92]

### Bioactive Ions

Bioactive metal ions, and in particularly calcium (Ca^2+^) [93], zinc (Zn^2+^) [94], magnesium (Mg^2+^) [95], and copper (Cu^2+^) ions [96], have been demonstrated to enhance bone regeneration through various mechanisms, such as promoting osteoblast differentiation and mineralization, facilitating angiogenesis, and regulating the expression levels of osteogenesis-related genes. Among these, bone tissue is rich in Ca^2+^, which plays a pivotal role in bone remodeling. Ca^2+^ not only facilitates the secretion of BMP-2 for osteoblast bone formation, but it also triggers the calcium sensing receptor for inflammatory suppression [97]. In addition, as a cofactor of some enzymes (including alkaline phosphate and collagenase), the Zn^2+^ present within the bone matrix can boost the bone metabolism and promote biomineralization [98, 99]. Furthermore, Mg^2+^ can improve the cell viability and differentiation of osteoblasts by activating the PI3K/Akt signaling pathway [100], while Cu^2+^ has been reported to strengthen collagen fibers and facilitate biomineralization [101]. In the context of vascularization, silicone ions (Si^4+^) can promote angiogenesis and induce the formation with vascularized bone by regulating the expression levels of angiogenic cytokine receptor-related genes and activating downstream signal transduction [102]. The osteogenic function of any bioactive metal ion depends on its concentration at the lesion sites, especially considering that these ions possess different toxicities and effective concentration. Therefore, local concentrations of bioactive ions play crucial roles in modulating new bone formation, while an excess can trigger bone loss and promote an abnormal bone metabolism. Thus, although small-molecule drugs are more stable than GFs, they require long-term delivery to meet bone reconstruction requirements. Thus, a bio-platform aimed at delivering the optimal concentrations of bioactive ion delivery would be preferred for bone regeneration over direct administration. Table 3 summarizes some of the latest therapeutic metal ions used bone regeneration.

**Table 3:** Representative bioactive ions applied in bone regeneration

Bioactive ion	Main bioactivity	Bone repair	References
Ca^2+^	Promote biomineralization	Cranial defect repair	[18]
	Promote osteogenic differentiation by activating the osteogenesis-related signaling pathway	Periodontal bone repair in diabetic rats	[93]
	Promote BMSCs osteogenic differentiation and angiogenesis	Cranial defect repair	[103]
Cu^2+^	Promote endothelial cell function and osteogenic differentiation of MSCs	Cranial defect repair	[19]
		Tendon-to-bone repair	[96]
Si^2+^	Promote immunomodulation and angiogenesis	Infected femoral bone defect repair	[104]
Si^4+^	Promote osteogenic differentiation	Tibia defect repair	[105]
	Promote BMSCs osteogenic differentiation and angiogenesis	Cranial defect repair	[103]
Mg^2+^	Accelerate stem cell differentiation toward osteogenesis	Femoral defect repair	[106]
		Cranial defect repair	[95]
	Regulate the bone metabolism and immune microenvironment to accelerate osteogenesis	Cranial defect repair	[107]
	Promote mouse embryo osteoblast precursor cell osteogenesis	Critical cranial defect repair	[108]
	Promote angiogenesis	Cranial defect repair	[109]
	Promote osteogenesis and angiogenesis by activating osteoblasts and endothelial cells	Osteoporotic bone defect repair	[110]
Zn^2+^	Promote biomineralization and stem cell differentiation	Periodontal bone repair	[94]
	Promote macrophages M2 polarization and osteo-induction	Infected bone repair	[30]
Lithium ions (Li^+^)	Relieve inflammatory reactions; Promote angiogenesis and osteogenesis	Diabetic femoral repair	[111]
	Promote nerve-bone regeneration by activating the Wnt/GSK-3*β*/*β*-catenin signaling pathway	Tibia defect repair	[112]
Strontium ions (Sr^2+^)	Enhance the osteoblast proliferation and differentiation capabilities; Exhibit a positive impact on the mitogenic signaling pathway	Cranial defect repair	[77]
	Improves hMSCs viability and osteogenic differentiation, inhibit osteoclast differentiation and viability	Femoral defect repair	[113]
Cobalt ions (Co^2+^)	Promote angiogenesis by upregulating CD31, VEGF, and HIF-1*α* expression to facilitate osteogenesis	Cranial defect repair	[114]

### Stem Cells and Extracellular Vesicles

Stem cell-based therapeutics show great promise for use in bone regeneration. More specifically, tissue deficiencies in the lesions can significantly impede the tissue remodeling process, and so the provision of exogenous stem cells can expedite tissue regeneration within a short time. In this context, MSCs have been used to enhance bone repair in pre-clinical models and clinical trials due to their ability to undergo self-renewal and differentiation toward osteogenesis [115]. In addition, MSCs are known to secrete cytokines, GFs, and metabolites via paracrine interactions to accelerate bone formation. Compared to MSCs, adipose-derived stem cells (ASCs) have become a more attractive cell source because of their minimally invasive acquisition approaches, greater abundance, and high production levels [116]. Studies have shown that ASCs can promote the density and formation of bone tissues for osteoporosis by upregulating Forkhead Box P1 (FOXP1) expression levels [117, 118]. However, stem cell therapies are commonly limited by a poor cell viability and engraftment. In recent years, an increasing number of studies have demonstrated that cell-secreted EVs play a key role in tissue regeneration by regulating the cell behavior and facilitating intercellular communication; as a result, they have become a promising alternative for cell therapy [119, 120]. EVs encompass exosomes (Exos) and microvesicles, which contain a diverse array of bioactive molecules, such as proteins, and nucleic acids [121]. Furthermore, BMSC-derived Exos (BMSC-Exos) can boost osteogenesis and angiogenesis. Indeed, in vitro tests have demonstrated that human BMSC-derived EVs enter the osteoblasts and release osteogenic microRNA (miRNA) by endocytosis, thereby upregulating the expression levels of osteogenesis-related genes and enhancing differentiation [122]. In addition, nidogen 1-enriched BMSCs-derived EVs (EV-NID1) are known to promote the migration of rat arterial endothelial cells (RAECs) and the formation of tubular structures by decreasing the adhesion strength between the RAECs and the extracellular matrix (ECM), finally facilitating angiogenesis to support bone regeneration [123]. Simultaneously, osteoclast-derived EVs with abundant secreted thrombin cleaved phosphoprotein 1 can enhance MSCs differentiation toward osteogenesis by activating the TGF-*β*1/Smad family member 3 (TGF-*β*1/Smad3) signaling pathway [124]. In addition, EVs can not only be customized to carry specific molecules that enhance osteogenesis, such as GFs, miRNAs, or signaling proteins, but also be engineered using various techniques, such as genetic modification, surface modification and loading of therapeutic agents [125, 126]. Engineered EVs hold great promise for treating bone defects, fractures, and diseases like osteoporosis. As a cell-free therapeutic technique, the application of EVs avoids the drawbacks related to losses in cell viability or the triggering of an immune response. Moreover, nanosized EVs facilitate administration and diminish vascular occlusion. However, two key challenges remain, namely the difficulties involved in obtaining large amounts of EVs for clinical applications, and their rapid clearance at lesion sites. Similar to the delivery of MSCs using biomaterials, the delivery of EVs via similar means has emerged as a promising tool for overcoming the low retention rates resulting from direct administration. Table 4 outlines bioactivities of various representative stem cells and EVs for use in bone regeneration.

**Table 4:** Representative stem cells and EVs applied in bone regeneration

Stem cells and EVs	Main bioactivity	Bone repair	References
BMSCs	Facilitate in situ bone formation by promoting proliferation, migration, and differentiation of exogenous BMSCs	Femoral defect repair in osteoporotic murine	[15]
Hypoxic EVs (BMSC-derived)	Facilitate cell proliferation, differentiation, and biomineralization via the phosphatidylinositide 3-kinase B pathway	Cranial defect repair	[16]
	Promote angiogenesis and couple with osteogenesis	Grafted tendon-bone tunnel healing	[127]
Normal glucose cultured BMSCs-derived EVs	Promote cell proliferation and migration, hinder cell apoptosis and facilitate BMSCs osteogenic differentiation	Diabetic cranial defect repair	[17]
Small EVs	Enhance osteogenesis by inhibiting M1 macrophage polarization (immunomodulation)	Osteoporotic tendon-to-bone healing	[128]
M2 macrophage-derived EVs	Relieve inflammatory reactions and promote osteogenesis	Diabetic alveolar bone repair	[129]
Periodontal ligament stem cells (PDLSCs)-derived Exos	Promote BMSCs osteogenic differentiation to accelerate new bone formation	Alveolar bone defects repair	[130]
Dental pulp stem cells-derived Exos (DPSCs-Exos)	Enhance osteogenic differentiation of pre-osteoblasts	Cranial defects repair	[131]
Human adipose-derived stem cells-derived Exo (hASCs-Exo)	Upregulate the expression levels of bone-related genes, such as ALP, OCN, and RUNX2	Knee osteochondral defects repair	[132]
Human umbilical vein endothelial cell-derived Exos (HUVECs-Exos)	Exos overexpressing PD-L1 boost the MSCs osteogenic differentiation of MSCs	Fracture healing	[133]
BMSCs-derived Exos	Exos with abundant *β*-catenin (CTNNB1) promote the osteogenic differentiation	Alveolar bone defect repair	[134]
Human adipose mesenchymal stem cells-derived Exos (hASCs-Exos) enriched with miR-375	Promote the osteogenic differentiation of BMSCs	Cranial defect repair	[135]
Schwann cell-derived Exo (SC-Exos)	Promote innervation, immunoregulation, vascularization, and osteogenesis to enhance bone formation	Cranial defect repair	[136]

### Genes

Gene therapies have been gained increasing attention for repairing bone defects in recent years. Gene drugs, including miRNA, small interfering RNA (siRNA), and CRISPR-Cas9 have been employed to continuously express therapeutic genes, effectively addressing some of the limitations associated with other bioactive molecules, such as high cost and short half-time of GFs, and the reduced viability of stem cells. miRNA is a class of small noncoding RNA molecules that play a crucial role in regulating gene expression through binding to the complementary sequences on the target mRNA [137]. Pro-angiogenic miR-21 can upregulate MAPK signaling pathway and PID-HIF1-TF pathway, thereby enhancing angiogenesis both in vitro and in vivo. Concurrently, pro-osteogenic miR-5106 significantly promotes differentiation of BMSCs into osteogenic line via upregulating the expression osteogenesis-related genes (e.g., ALP, RUNX2, and OCN). In vivo, miR-5106 can also accelerate bone formation via enhancing the mineralization rate within bone defects [138]. In addition, RNA interference-mediated gene silencing has been extensively utilized for bone defect healing through modulating the expression of cytokines-related osteogenesis and angiogenesis [139]. siRNA-based gene therapy offers greater precise and predictable than miRNA therapy due to its complete complementary with the targeting miRNA sequences [140]. For example, the soluble interfering VEGF receptor 1 (siFlt-1) can inhibit sFlt-1 expression and then upregulate VEGF, ALP expression for repairing skull defects. Furthermore, the small interfering p75 neurotrophic factor receptor (sip75^NTR^) can knock down the p75^NTR^ genes to facilitate neurogenic bone regeneration via promoting glial fibrillary acidic protein and *β*-nerve growth factor (*β*-NGF) expression [141]. However, direct administration of genes to the injured bone is limited by several factors, including dilution of biological fluids, apoptosis of transfected cells, enzymatic degradation, and low bioavailability [142, 143]. As a result, effective gene delivery remains a great challenge in achieving high therapeutic efficacy at both cellular and tissue levels. To address these challenges, both viral and non-viral carriers have been widely used for gene delivery to the injured bone tissues. Non-viral vectors, in particular, have demonstrated lower toxicity, greater immunological safety, cost-effectiveness, and higher transfection efficiency compared to viral carriers, while also enabling modulation of gene expression across in vitro, in vivo, and ex vivo environment [144]. Currently, nano- and micromaterials, as well as hydrogel and scaffolds, have been explored as platforms to enhance the therapeutic outcomes of gene delivery strategy. Table 5 summarizes the latest gene drugs applied in bone regeneration.

**Table 5:** Representative genes applied in bone defects healing

Genes	Main bioactivity	Bone repair	References
miR29c	Reduce Dickkopf 1 expression, inhibit the Wnt pathway, and promote the osteogenic differentiation of BMSCs	Cranial defect repair	[145]
miR335-5p	Promote BMSCs viability and reduce BMSCs apoptosis, promote ALP expression and calcium nodular deposition	Bone defect in steroid-associated osteonecrosis	[146]
miR-26a	Promote osteogenesis and angiogenesis via suppressing GSK-3*β* signaling and enhancing the development of blood vessels	Critical-sized cranial defect repair	[147]
		Femoral defect repair and Osteoporosis prevention	[148]
WW domain-containing E3 ubiquitin protein ligase 1 (Wwp1) siRNA	Induce Wwp1 silence to promote bone regeneration	Fracture healing	[149]
siRNA (siDcstamp)	Promote osteogenesis and hinder preosteoclast fusion and bone resorption via interfering Dcstamp mRNA expression	Osteoporotic vertebral trabecular bone defect repair	[150]

## Hydrogel-Based Delivery Strategies for Bone Regeneration

### Conventional Delivery

Hydrogels possess unique swelling properties that enable them to act primarily as a medium for facilitating the delivery of bioactive molecules through synergistic effects between the intrinsic swelling behavior and time-dependent degradation off the hydrogel platform. Swollen hydrogels promote molecular exchange by absorbing nutrients from the external environment to achieve the sustained release of bioactive molecules [151]. For a biodegradable hydrogel matrix, the swollen state is only short-lived, and the hydrogel can be degraded with the assistance of the in vivo microenvironment [152]. This delivery system offers a straightforward strategy for maintaining a stable molecular accumulation in vivo, thereby strengthening both the bioavailability and biosafety profiles of the bioactive molecules. Compared to trauma and surgery-induced common bone injuries, bone defects caused by bone tumors, osteoporosis, and diabetes exhibit more complex and specific characteristics. To overcome these challenges, therapeutics combining bioactive molecules for bone therapy and drugs for disease treatment are usually applied. Bone injury can be divided into four classes based on the location of the bone tissue, namely cranial defects, femoral defects, periodontal bone defects, and osteoporosis with systematic bone loss. In this section, the conventional application of hydrogels as a platform to deliver bioactive molecules in such scenarios is introduced.

#### Cranial Defect Repair

The cranial tissue plays an important role in preventing damage to the ultrasoft brain tissue. As a major component of the central nervous system, the brain tissue focuses on modulating vital functions. Generally, cranial defects are a clinical challenge that can arise from a variety of causes, including congenital dysraphism, skeletal anomalies, acquired injuries resulting from trauma [153, 154], craniotomy surgery [155], and infection [156]. Such injuries are of particular concern since skull loss can trigger imbalances in the cerebrospinal fluid circulation [157]. Conventional treatment approaches for cranial defects in a clinical setting include artificial/natural bone grafts and titanium/polyether ether ketone substitutes. However, the limited availability of donor grafts, poor osteointegration, and the low osteoconductivity characteristics of such substitutes can hinder the self-healing of bone tissue [158, 159]. Given that bioactive molecules are capable of facilitating stem cell differentiation and bone regeneration, the introduction of these molecules into hydrogels to achieve sustained release is highly favorable. Moreover, adjunctive measures, such as hypoxia, drugs, and other bioactive molecules, are commonly used to promote cranial defect repair.

**Fig. 2:**
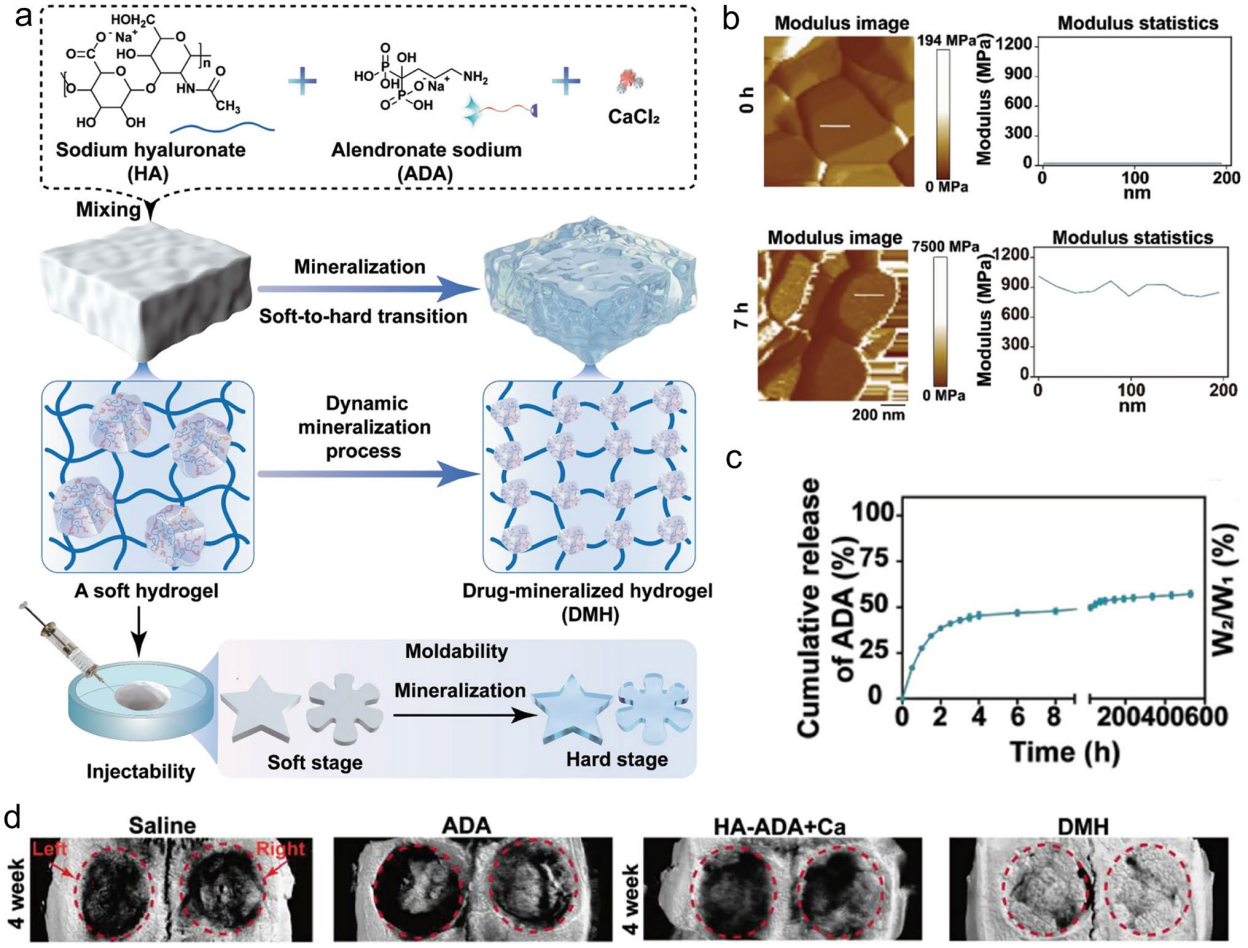
**a** Illustration of DMH hydrogel fabrication and dynamic mineralization from the soft stage to the hard stage. **b** Young’s modulus and modulus statistics of the DMH hydrogel at 0 and 7 h. **c** ADA release curves from the DMH hydrogel in PBS solutions at 37 °C. **d** CT images of the cranial defects after treatment with the DMH hydrogel and the control groups on week 4 (n = 4). **a**–**d** Reproduced with permission. Copyright 2023, John Wiley and Sons [80]

**Fig. 3:**
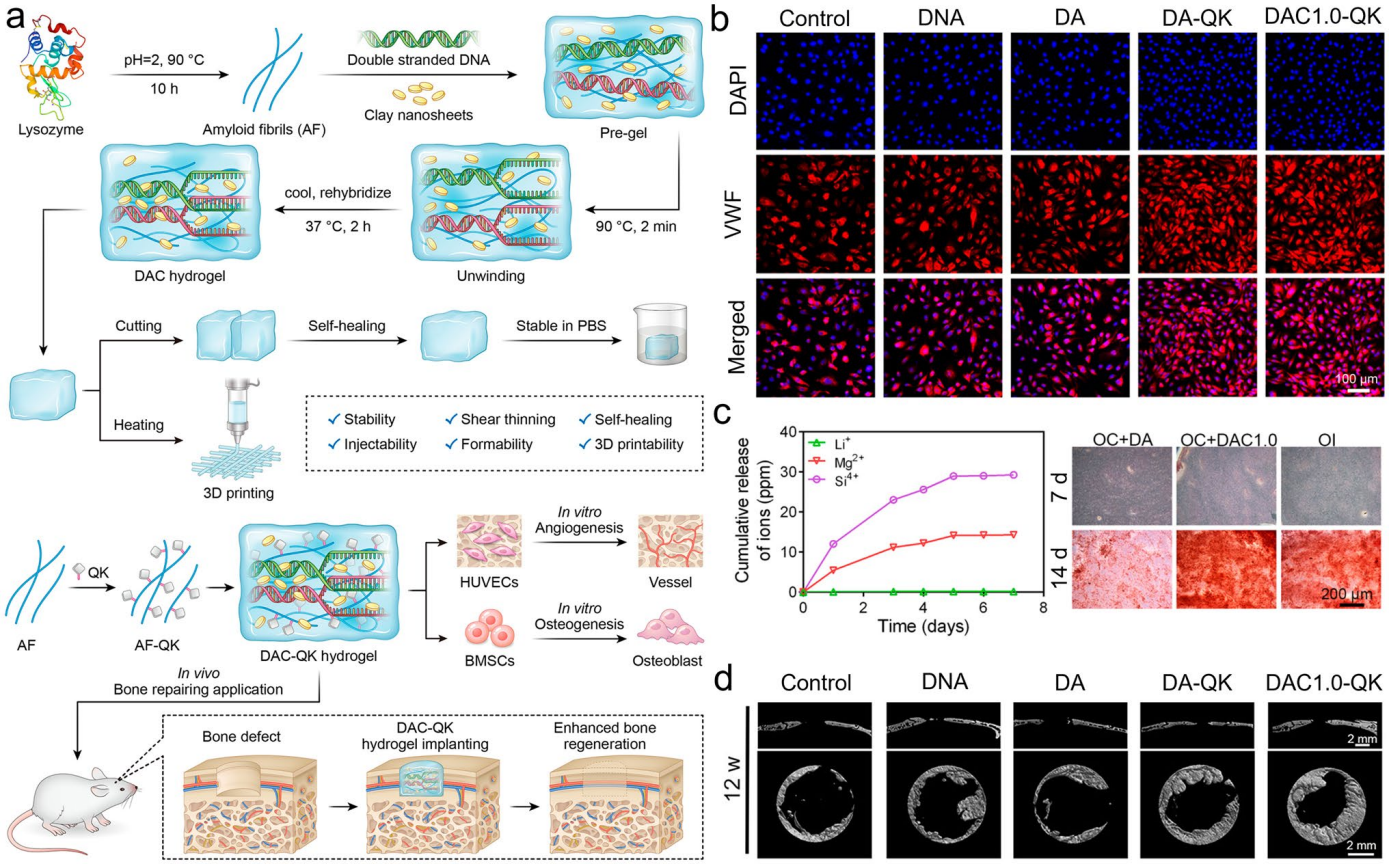
**a** Schematic illustration of the preparation, properties, and cranial defects repair capability of the DAC hydrogel. **b** Immunofluorescence staining of HUVECs, and the VWF protein (stained with red color), and the nuclei were stained with blue color, respectively. **c** Release curves of the bioactive ions from the hydrogel at different times and alizarin red staining images of BMSCs cultured with the extract medium for 7 and 14 days. **d** Cross-sectional and longitudinal micro-CT images of cranial defects treated with the hydrogels. **a**–**d** Reproduced with permission. Copyright 2023, American Chemical Society [162]

As a vital composition of bone tissue, Ca^2+^ plays a critical role in bone remodeling. With this in mind, Zhang et al. [18] incorporated multifunctional magnesium ascorbyl phosphate (MAP) into a gelatin methacrylate (GelMA) hydrogel as an exogenous supplement to phosphorus-containing compounds. In vitro tests showed that MAP removed ROS to protect the BMSC viability, while also facilitating Ca^2+^ uptake to accelerate BMSC biomineralization in vitro. The results of microtomography (micro-CT) indicated that the bone defects treated with the MAP hydrogel (32.74 ± 0.62%) exhibited a significantly higher healing efficiency than the control group (7.08 ± 0.29%). In addition, inspired by the natural mineralization process, a therapeutic combination based on Ca^2+^ and bioactive molecules has also been developed. More specifically, Liu et al. [80] reported the incorporation of an ADA-CaCl_2_ complex into a sodium hyaluronate (HA) hydrogel. The HA regulated the dynamic mineralization of the ADA-Ca^2+^ complex, conferring the hydrogel with a spontaneous dynamic mineralization behavior and moldability in the early stages (Fig. 2a, b). The sustained release of ADA lasted for 22 days with gradual degradation of the drug-mineralized hydrogel (DMH), thereby promoting cranial defect repair (Fig. 2c, d). Similarly, Zou et al. [160] developed a hydrogel containing the PTH peptide PTH(1–34) and nano-hydroxyapatite (nHAp) via electrostatic interactions between chitosan (CS) and sodium alginate (SA). In vitro tests showed that this hydrogel upregulated the expression levels of RUNX2, ALP, and osteopontin (OPN), in addition to boosting the osteogenic differentiation of rat BMSCs. Compared to rats treated with the CS/SA hydrogel alone, the Gel-nHAp-PTH hydrogel facilitated efficient bone regeneration. Based on the freeze-drying technique, Li et al. [161] prepared a CS/silk cryogel containing silver (Ag) and Sr-doped HAp for cranial defect repair. This cryogel exhibited a good resilience and flexibility, ensuring a sufficient mechanical strength for bone regeneration. Importantly, the sustained release of the Ag and Sr ions conferred long-term antibacterial properties and osteoinductivity to the cryogel. In this delivery system, both Ag and Sr ions were introduced into the nHAp crystal lattice, thereby avoiding any cytotoxicity related to the burst release of ions to ensure long-term ion delivery. In addition, dynamic hydrogels with reversible mechanics that mimic the biophysical cues of the in vivo ECM have also been reported. For example, Yang et al. [162] proposed a nanoengineered DNA dynamic hydrogel (DAC) based on supramolecular co-assembled interactions between clay NSs, QK peptide-grafted amyloid fibrils, and DNA strands (Fig. 3a). The combination of amyloid fibrils and clay NSs endowed the DNA hydrogel with the good mechanical properties and stability of a dynamic hydrogel. The QK peptide was sustainably released from the hydrogel matrix, facilitating vascularization (Fig. 3b), while the Si^4+^ and Mg^2+^ components present in the clay NSs were released to enhance the differentiation of BMSCs toward osteogenesis (Fig. 3c). In vivo rat cranial defect experiments also demonstrated that this dynamic DNA hydrogel could successfully achieve vascularized bone repair (Fig. 3d). In another study, Mao et al. [163] constructed a shape-memory silk fibroin/magnesium oxide (SF/MgO) composite scaffold using the trimming technique, which easily matched irregular cranial defects. During degradation of the MgO particles within scaffold, Mg^2+^ was gradually released to modulate cell functions and upregulate the expression levels of osteogenesis-related genes. Furthermore, BMPs have been clinically approved by the US FDA for bone treatment, and have been widely incorporated into hydrogel systems to facilitate bone and cartilage formation. For example, Pal et al. [14] developed a collagen/elastin-like polypeptide (ELP) hydrogel with an interpenetrating network that encapsulated BMP-2, doxycycline (Doxy), and 45S5Bioglass. The results of in vitro studies demonstrated that the collagen-ELP hydrogel promoted HAp particles deposition after culture for 21 days. After implantation into a rat possessing a critical-sized cranial defect, the hydrogel promoted mineralization, as evidenced by scanning electron microscopy (SEM), micro-CT observation, and histological analysis. Moreover, Liu et al. [164] reported a poly(lactic-*co*-glycolic acid)/poly(2-(methacryloyloxy)ethyl)dimethyl-(3-sulfopropyl) ammonium hydroxide hydrogel (PLGA/PSBMA) scaffold for the delivery of rhBMP-2 to repair critical-sized cranial defects. PSBMA, with its oppositely charged structure, was found to enhance the well-integrated mineralization of calcium phosphate (CaP) within the PLGA/PSBMA scaffold. rhBMP-2 exhibited a high affinity for the mineralized scaffold, which enabled the gradual release of rhBMP-2 from the mineralized scaffolds (i.e., 1.7% release within 35 days) to facilitate defect repair. This scaffold also provided a platform for the delivery of rhBMP-2 at an ultralow dose (150 ng per scaffold). Additionally, hypoxia-preconditioned BMSCs-derived biglycan(Bgn)-rich EVs have been demonstrated to exhibit a superior therapeutic efficacy to pure BMSCs-derived EVs. In this context, Deng et al. [16] developed a hypoxic EVs (Hypo-EVs)-loaded poly(ethylene glycol)(PEG)/polypeptide co-polymer hydrogel for cranial defect regeneration (Fig. 6a, b). This bioactive hydrogel was able to continuously deliver Hypo-EVs for up to day 21 (~ 87.6% accumulative release; see Fig. 6c) to significantly promote osteoblast proliferation, differentiation, and biomineralization via the phosphatidylinositide 3-kinase/protein kinase B signaling pathway. In vivo experiments revealed that the hydrogel substantially boosted the repair of 5-mm rat cranial defects. Bgn is known to play a crucial role in maintaining the activity and function of osteoblasts. Moreover, hypoxic Exos can promote angiogenesis, further accelerating new bone formation to repair bone defects. With these considerations in mind, Zhong et al. [165] produced hypoxia-triggered exosome mimetics (HY-EMs) to enhance the in vitro and in vivo pro-angiogenic abilities of hypoxia-treated BMSCs using the serial extrusion method. The EMs yield was found to be ~ 10 times higher than that of the Exos. Notably, with the sustained release of HY-EMs from the subcutaneously implanted HY-EMs-GelMA hydrogel scaffold, the number of blood vessels increased by 186%. The effectiveness of vascularized osteogenesis in a critical-sized cranial defect model is mainly reflected by the increased bone volume (120%) and vessel number (175%) in the regenerated bone.

In addition, the intrinsic characteristics (including shape adaptivity, stiffness, and degradation) of conventional hydrogels have significant implications in the context of cranial regeneration. For example, Zou et al. [166] incorporated a metal-phenolic network of tannic acid (TA) and Fe_3_O_4_ NPs into regenerated SF (RSF) to generate a fast- gelling and shape-adaptive RSF/TA/Fe_3_O_4_ hydrogel that could respond to a static magnetic field (SMF). The hydrogel adapted automatically to the shape of the defect, seamlessly binding to the surrounding tissue, and effectively covering the defect. Additionally, the incorporation of Fe_3_O_4_ NPs endowed this hydrogel with tunable biodegradation characteristic, allowing adjustment of the RSF/TA/Fe_3_O_4_ hydrogel to achieve a biodegradation rate that was equivalent to the rate of bone formation, and reaching a safe does of 48.12 ± 2.67 μg mL^−1^ on day 28. In contrast to conventional therapeutics, the optimal osteogenesis capability of the Fe_3_O_4_ NPs must be activated using an SMF, further triggering the cGMP/PKG/ERK signaling pathway to facilitate osteoblast cell differentiation. Since the mechanical properties of a hydrogel can influence the migration and differentiation of MSCs during the process of bone healing, the mechanical strength of the hydrogel-based delivery system must be tuned by incorporating various inorganic agents, modifying the chemical composition of the hydrogel matrix, or controlling the delivery of bioactive molecules, to ultimately provide an ECM-like microenvironment for accelerated bone regeneration. In this context, Ren et al. [52] rationally designed a PRF-based hydrogel by combining polydopamine (PDA)-modified silicone dioxide (SiO_2_) nanofibers for cranial regeneration. With an increase in the nanofiber content, the PRF hydrogel became stiffer, hindering its rapid decomposition and maintaining the sustained release of autologous growth factors to achieve long-term osteogenesis. Moreover, the reinforced hydrogel mimicked the ECM to promote osteoblast differentiation via the Yes-associated protein (YAP) signaling pathway to finally repair cranial defects. In addition, some studies have demonstrated a direct connection between skeletal stem cells (SSCs) and bone regeneration due to the proliferation of SSCs at lesion sites after bone injury [167]. However, SSCs tend to possess short lifespans, and so when used alone, they are unable to support complete bone healing [168]. Considering that soluble factors, such as neurotrophic supplements (NSs), can promote SSCs expansion, Zhang et al. [169] incorporated the optimized NSs into a three-dimensionally (3D-) printed hydrogel scaffold as a cell culture system to trigger MSCs expansion in vitro, and to promote the full-thickness repair of critical-sized cranial defects. According to their single-cell RNA analysis results, this 3D-printed scaffold culture system also promoted myxovirus resistance-1^+^ SSC recruitment and expansion in situ. Overall, their system represented a highly efficient strategy for repairing critical-sized bone defects. Moreover, it has been reported that the osteogenic growth peptide-like C-terminal fragment (YGFGG) can be used to promote osteoblast proliferation and differentiation [170]. However, short half-life and poor tissue retention capability of this fragment require improvement. Moreover, considering that virus-like particles (VLPs) can be regarded as carriers for peptide delivery, they are commonly used as nanocages to deliver various viral capsid proteins. In this context, Shen et al. [171] constructed a CS scaffold composed of lysozyme and YGFGG-decorated cowpea chlorotic mottle virus nanoparticles (CCMV-YGFGG) for the reparation of infected bone defects. Lysozyme was released to reduce the inflammatory response triggered by the infection, while the gradual delivery of CCMV-YGFGG promoted osteogenesis for new bone formation.

Bone regeneration therapeutics that are used in healthy animals have also been applied to animals suffering from underlying diseases (e.g., diabetes mellitus (DM) and osteoporosis), often leading to unsatisfactory results. DM is a metabolic endocrine disease that induces many complications that affect the body [172]. DM can also cause osteoporosis, leading to increased fracture rates, and the requirement to repair bone defects. In a high-glucose environment, the bone regeneration process is prolonged, and bone construction is weakened. To demonstrate this, Yang et al. [17] compared the bone regeneration efficacies of high-glucose-cultured BMSCs-derived EVs (HG-EVs) and normal glucose cultured BMSCs-derived EVs (NG-EVs) using a sustained release system, which was developed by utilizing bioactive PDA to assist the encapsulation of EVs by gelatin (Gel)/HA/nHAp scaffolds via 3D printing (Fig. 7h, i). Their results showed that both the HG-EVs and NG-EVs released from the hydrogel scaffold facilitated osteogenic differentiation and cell proliferation, although the therapeutic effect of the HG-EVs was weaker than that of NG-EVs. Thus, under diabetic conditions, endogenous EVs were not suitable for application in bone defect regeneration. PDGF-BB is typically used to treat various complications induced by DM and is known to restore cell proliferation and osteogenesis under high-glucose conditions. In this context, Li et al. [173] utilized the nano-strengthening effects of the LAPONITE^®^ (Lap) nanoclay to design a GelMA-based PDGF-BB delivery platform (PDGF@Gel-Lap) for treating cranial defects under diabetic conditions. PDGF-BB was gradually released from the hydrogel, while bioactive ions (e.g., Si^4+^, Mg^2+^, and Li^+^) were also released from the nanoclay. According to the results obtained in diabetic rats with bilateral cranial defects, the PDGF@Gel-Lap hydrogel exhibited the largest coverage area of new bone compared with the PDGF@Gel and Gel-Lap hydrogel-treated groups. In addition, with the consideration that pathological DM triggers chronic inflammatory reactions and hinders biomineralization, Xu et al. [174] reconstructed a mild microenvironment by fabricating a 3D-printed GelMA composite bioscaffold using Sr-loaded mesoporous bioactive glass nanoparticles (Sr-MBGNs). This scaffold was able to effectively deliver Sr, Ca, and Si ions, further enhancing the immunomodulatory, angiogenic, and osteogenic behaviors of the scaffold.

Osteoporosis is one of the most disabling conditions in the aging population, leading to bone loss and microarchitectural deterioration [175, 176]. As a result, patients suffering from osteoporosis are highly sensitive to even small traumas due to their high bone fragility and fracture incidence [177]. The self-healing of osteoporotic bone defects and fractures therefore challenging because of the impaired bone regeneration capacity under the chronic inflammatory conditions induced by osteoporosis [178]. Thus, due to the aging population, the social and economic burdens associated with osteoporotic fractures are increasing worldwide [179]. With this in mind, Sun et al. [12] developed a bioactive composite hydrogel for the repair of cranial defects, which was based on the incorporation of short chain CS and nHAp into a tetra-armed PEG (tetra-PEG) hydrogel (PEG/nHAp/CS, PHC) and the subsequent encapsulation of PTH. The composite hydrogel exhibited a strong immunomodulatory performance through its simultaneous enhancement of M2 macrophage polarization and inhibition of M1 macrophage polarization, which was achieved via the TLR4/NF-κB signaling pathway. Moreover, the continuous release of PTH enhanced the osteogenic capability of this system via the cAMP/PKA/CREB signaling pathway. Osteoporotic animal experiments further demonstrated that this hydrogel could accelerate the reconstruction and healing of cranial defects. To decrease the dosage required to achieve an optimal efficacy, Echave et al. [180] reported a BMP-2-loaded Gel-based scaffold, which was strengthened by adding osteoconductive calcium sulfate or HAp. Upon the application of this system for osteoporotic cranial defect repair, in vitro tests demonstrated that the hydrogel scaffold containing 7.5% (m/v) ceramic compounds exhibited higher Young’s moduli (i.e., 179 kPa for CaSO_4_ and 75 kPa for HAp) than that of the pure Gel scaffold (i.e., 48 kPa). These reinforced scaffolds allowed the sustained release performance of BMP-2 at a relatively low dosage (600 ng BMP-2 per scaffold) in comparison with common doses with 2–15 µg in previous studies. The results indicate the desirability of employing hydrogels for the treatment of cranial injuries resulting from trauma, infection, chronic inflammation, and tumor, especially in the case of irregular bone defects.

#### Repair of Femoral/Tibial Defects

**Fig. 4:**
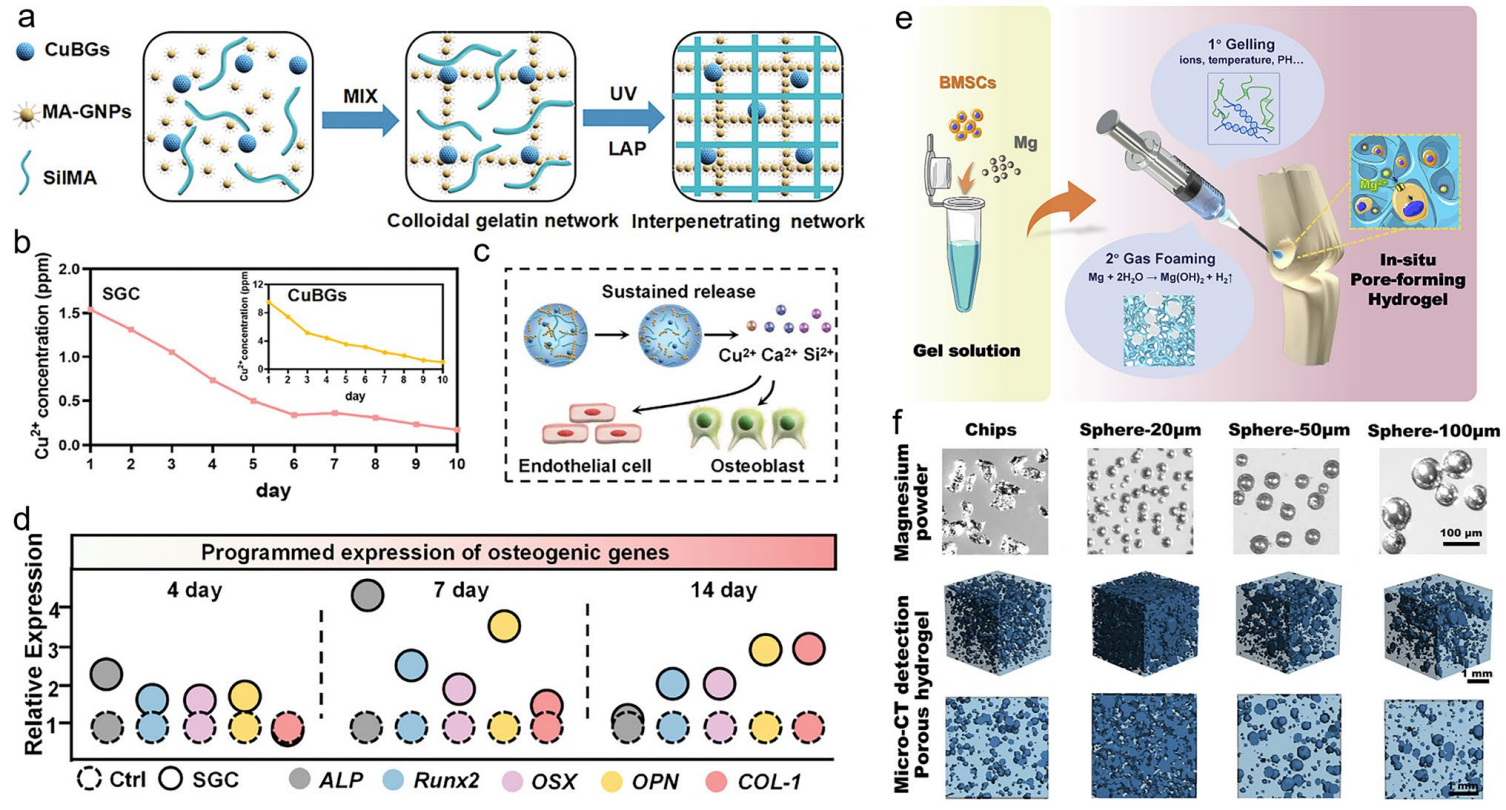
**a** Synthesis of the interpenetrating SGC hydrogel network by dual-photo-crosslinking interactions. **b** Cu^2+^ release curves and schemes for the CuBG and SGC hydrogels over a period of 10 days. **c** Mechanisms of angiogenesis and osteogenesis after being treated with SGC hydrogel. **d** The relative expression of osteogenesis-related genes at different time. **a**–**d** Reproduced with permission. Copyright 2023, John Wiley and Sons [104]. **e** Preparation of a BMSCs and Mg^2+^-loaded microporous hydrogel using an in situ gas foaming strategy. **f** Mg powders of different sizes endowed the hydrogel with different porous structures. **e**, **f** Reproduced with permission. Copyright 2020, Elsevier [106]

The femur is the longest and thickest bone tissue in the human body and is located in the lower limb of the quadrupeds. Following an injury, femoral defects can cause femoral fractures and femoral head necrosis. This section reviews the exploration of drugs and grafting products for the repair of femoral and tibial defects. More specifically, Hou et al. [78] fabricated a DFO-loaded tough silk nanofiber-based cryogel with an enhanced osteogenic bioactivity. The mechanical properties and release behavior of DFO were modulated by adjusting the silk nanofiber concentration, achieving a modulus of > 400 kPa, along with the gradual release of DFO over 60 days. This controlled modulation induced both osteogenic and angiogenic activities, promoting bone regeneration in rat femur defects and resulting in the accelerated regeneration of vascularized bone tissue. In another study, Fang et al. [181] developed an osteoconductive polyacrylamide (PAAm)/polyurethacrylate dextran hydrogel (PADH), which was subjected to in situ HAp nanocrystal mineralization for application in femoral condyle defect repair. Compared with the pure PAAm hydrogel, this hydrogel exhibited a high compressive strength (6.5 MPa) and fracture resistance (> 2300 J m^−2^). According to the in vitro tests, mineralized HAp promoted osteoblast adhesion, proliferation, and osteogenic differentiation. In a femoral condyle defect rabbit model, treatment with the HAp-PADH hydrogel led to the formation of highly mineralized bone tissue, suggesting that the hydrogel possessed a good osteoconductivity and osteointegration capability. Inspired by mussels, Liu et al. [182] fabricated an injectable hydrogel by incorporating PDA-decorated nHAp (PHAp) into an oxidized SA (OSA)/Gel hydrogel matrix using Schiff-base crosslinking for application in femoral condylar bone defect repair. Rheological measurements indicated that the gelation time of this hydrogel was 3–7 min, which is compatible with clinical applications. The incorporation of PHAp not only strengthened the mechanical strength of the hydrogel, but it also promoted the adhesion, proliferation, and osteogenic differentiation of BMSCs, further enhancing repair of the femoral defects. Recovery from infectious femoral injuries is significantly more challenging than from common injuries, mainly owing to the additional complications of bacterial colonization and secondary damage. To address this, Jian et al. [104] developed a methacrylated silk fibroin (SilMA) hybrid hydrogel (SGC) incorporating with Cu doped bioglass (CuBG) and methacryloyl-functionalized Gel NPs to heal infected femoral defects (Fig. 4a). This hybrid hydrogel could sequentially release Cu^2+^, Ca^2+^, and Si^2+^ from the CuBG system on demand (Fig. 4b, c), simultaneously conferring an antibacterial activity, immunomodulation, and bone reconstruction abilities to this hydrogel (Fig. 4d), while also effectively inhibiting infection and facilitating rapid femur defect repair. Furthermore, Jiang et al. [183] developed alginate/silk sericin/GO (Alg/Ser/GO) hydrogels via enzymatic crosslinking to repair irregular bone defects. This hydrogel exhibited a good bioimaging ability and controlled degradation properties, further improving the dual-release behavior of Ser and GO. More specifically, Ser and GO exhibited synergistic effects and promoted osteogenic differentiation and mineralization of the encapsulated rat BMSCs; Ser also promoted the M2 polarization and migration of macrophages. In vivo experiments revealed successful bone regeneration, suggesting that the Alg/Ser/GO hydrogel can be considered a promising biomaterial for healing irregular bone defects. Moreover, cell therapy holds great promise as an emerging therapeutic strategy for the treatment of femoral head necrosis. Tang et al. [106] used a gas-generation strategy to create pores by incorporating Mg particles into a cell-encapsulated hydrogel. Gradual degradation of the Mg particles generated H_2_ gas, leading to numerous pores within the hydrogel. At the same time, the Mg^2+^ produced during degradation of the Mg particles accelerated the differentiation of BMSCs in the osteogenic direction (Fig. 4e, f).

**Fig. 5:**
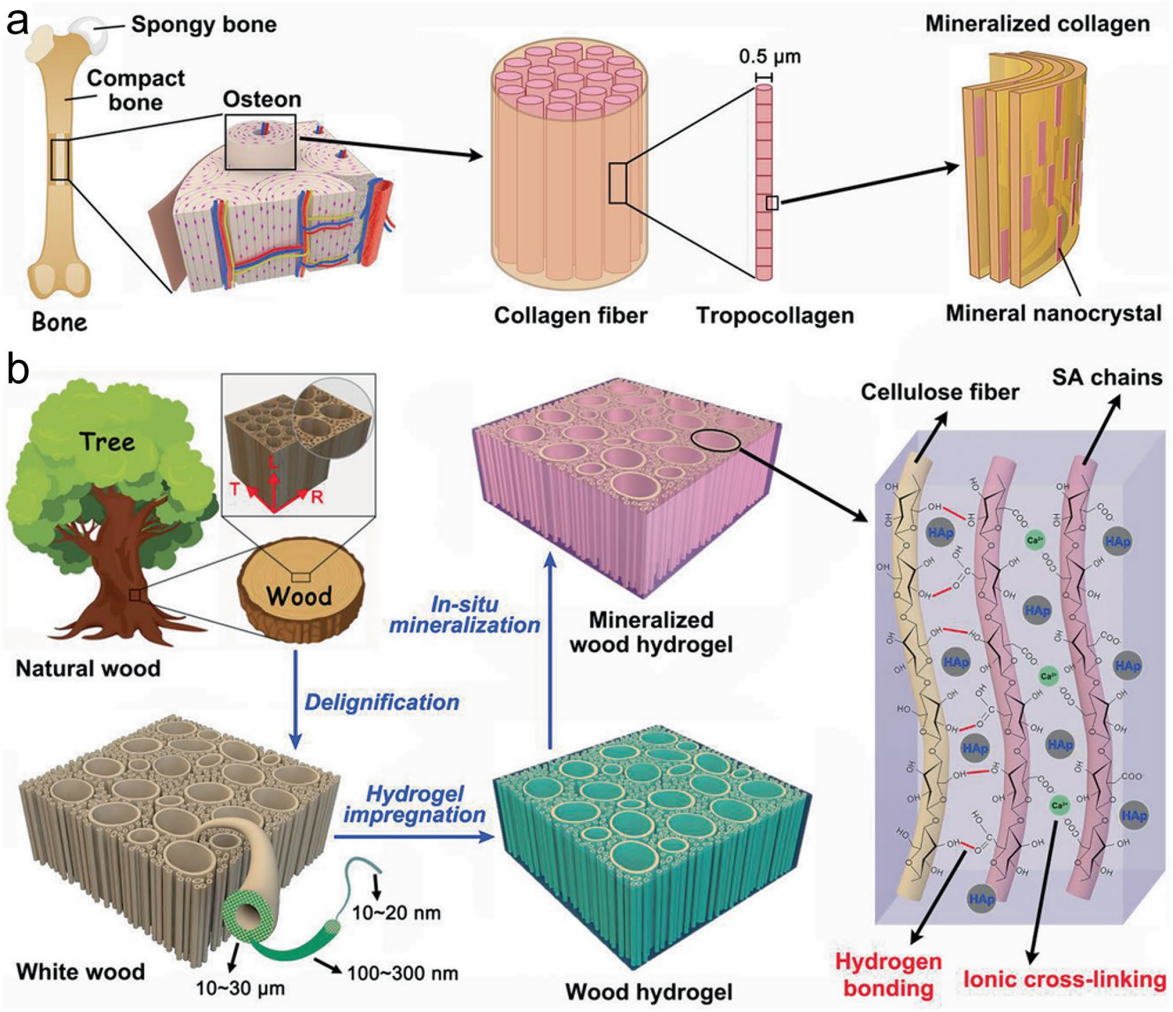
Schematic illustration of **a** the design strategy, **b** preparation, and microscopic structure of the mineralized wood hydrogel. Reproduced with permission. Copyright 2021, John Wiley and Sons [184]

Bone tissues usually exhibit directional functions, including damage tolerance, cell guidance, and substance exchange. Thus, the applications of traditional hydrogels are limited because of their isotropic structures, poor mechanical strengths, and osteoconductivity characteristics. To address this challenge, Wang et al. [184] fabricated a natural bone- and wood-inspired hydrogel composite by impregnating SA hydrogels into a white wood matrix with aligned cellulose fibril skeletons, followed by in situ nHAp mineralization and Ca^2+^-triggered ionic crosslinking of the SA hydrogel (Fig. 5). The well-aligned cellulose nanofibrils ensured that the hydrogel possessed an anisotropic structure in addition to a good mechanical strength, a high tensile strength (67.8 MPa), and a suitable elastic modulus (670 MPa), which were significantly higher than those of pure SA hydrogels. The addition of SA and nHAp into the porous wood matrix not only accelerated new bone formation at the scaffold-bone tissue interface, but it also promoted osteointegration to boost new bone growth within the scaffold. To build a pro-healing microenvironment for femoral defect repair under diabetic conditions, Xiang et al. [75] utilized macroporous silk gel scaffolds to deliver sitagliptin in situ. This system exhibited a high bioavailability, enhanced the recruitment of M2 macrophages, and showed osteointegration characteristics. In another study, Moradi et al. [88] integrated CS, GO, hydroxyethyl cellulose, and *β*-glycerol phosphate into an Atsttrin-loaded injectable hydrogel at physiological temperature. Upon increasing the amount of GO, the hydrogel presented a higher crosslinking density, along with the prolonged and sustained released of Atsttrin for the treatment of impaired fractures, especially femoral fractures healing under diabetic conditions. In addition, bioactive ions and stem cell-derived EVs have been delivered to promote diabetic bone repair. For example, Wu et al. [111] incorporated Li^+^-modified BG into a GelMA hydrogel for diabetic bone repair. The release of Li^+^, Si^4+^, PO_4_^3−^, and Ca^2+^ ions from the hydrogel was well-controlled and prolonged, indicating the suitability of this system for healing diabetic femoral defects. Although bone tissue possesses a highly regenerative potential, the healing of comprised femoral or tibial fractures remains a major challenge, with the rate of non-union prevalence reaching > 10% [185, 186]. By remodeling the metabolic microenvironment, Zha et al. [187] developed a 4-octyl itaonate (4-OI) and Cu^2+^-loaded Gel nanocomposite hydrogel (4-OI@Cu@Gel) for femur fracture healing. This hydrogel alleviated inflammatory reactions and promoted M2 polarization by the burst release of 4-OI, delivering Cu^2+^ to enhance glycolysis and osteogenic differentiation of the BMSCs, simultaneously facilitating angiogenesis, and finally achieving rapid femur fracture healing in mice. As mentioned above, osteoporosis is a bone disorder characterized by a low bone mineral density, a reduced bone strength, and an increased fragility, which ultimately increases the risk of fractures. Indeed, in China, approximately 2.3 million osteoporotic fractures occurred in 2010 [188]. Osteoporotic fractures are more difficult to repair owing to their weak skeletal strength, low bone density, poor trabecular microstructure, and limited bone formation capability compared to normal fractures [189]. Furthermore, osteoporosis is considered a chronic inflammatory disease, and the silent nature of this disease can lead to hospitalization and subsequent secondary health problems. As a potential treatment option, Song et al. [128] developed an ASCs-derived small EVs-loaded SA/HA hydrogel (MHA-sEVs) with a microporous aligned structure. This hydrogel allowed the sustained release of sEVs and exhibited an immunomodulatory function by inhibiting M1 macrophage polarization, ultimately enhancing bone regeneration. Recently, a number of novel therapeutics based on bioactive molecules and cellular biology have been developed for the treatment of osteoporosis [190], wherein the strategic design of combination therapies aimed at promoting bone growth has been demonstrated to hold potential for the improved management of osteoporosis. In addition, the advancement of simpler and more industrially scalable manufacturing techniques for regenerative approaches is crucial to facilitate efficient translation from the bench to the clinical environment.

Glucocorticoid-induced osteonecrosis of the femoral head (ONFH) is a serious disease in young men. ONFH results from an impaired blood supply, causing bone cell and bone marrow death, and leading to structural changes and collapse of the femoral head. This condition, which is associated with joint pain and dysfunction, often lacks an effective clinical treatment, necessitating total hip arthroplasty for many patients, which causes great pain and imposes a high financial burden on patients. Hence, the key to manage ONFH is to reverse the early stages of the condition and promote operative bone remodeling. In this context, Chen et al. [191] developed a BMSCs-derived Exos-loaded ECM-mimicking methacryloylated type I collagen hydrogel to sustainably release Exos and treat femoral head osteonecrosis. They found that the hydrogel incorporated with Li^+^-stimulated BMSCs-derived exosomes facilitated M2 polarization and osteogenesis to a greater extent than the hydrogel containing pure BMSCs-derived exosomes, as evidenced by in vivo experiments. In addition, Maruyama et al. [192] encapsulated lipopolysaccharides and TNF-*α*-treated MSCs (pMSCs) into an injectable hydrogel for ONFH repair. In vivo experiments revealed that the sustained release of pMSCs could facilitate angiogenesis and osteogenesis at femoral head lesion sites. Bone tumors, especially for osteosarcoma, are characterized by a highly malignant spindle cell sarcoma, which forms a tumor-like bone matrix [193]. Osteosarcoma generally appears in the epiphyseal ends of bones. Thus, although invasion and surgical procedures have been used to remove bone tumors, this process causes significant large bone loss, rendering it difficult to repair body defects without additional intervention. To address this issue, Liao et al. [194] designed a bifunctional hydrogel by incorporating gold nanorods (GNRs) and nHAp into a GelMA/methacrylated chondroitin sulfate (CSMA) hydrogel to treat residual tumors in a tibial animal model after surgery, and to promote new bone formation. The GNRs-based photothermal effect endowed the hydrogel with a good photothermal therapy (PTT) efficacy for residual tumor removal after surgery, and the addition of nHAp resulted new bone formation. In another study, Luo et al. [195] developed an oxidized SA/CS hydrogel containing cisplatin (DDP) and PDA-coated nHAp (PHA) via Schiff-base crosslinking. DDP was sustainably released from this hydrogel owing to the immobilization of PDA. The photothermal properties of PDA under NIR stimulation led to the significant ablation of 4T1 cells in vitro and inhibited tumor growth in vivo, further accelerating the delivery of DDP. In addition, the integration of PHA into hydrogels induced effective bone regeneration, although DDP was unable to significantly promote new bone formation.

#### Periodontal Bone Regeneration

**Fig. 6:**
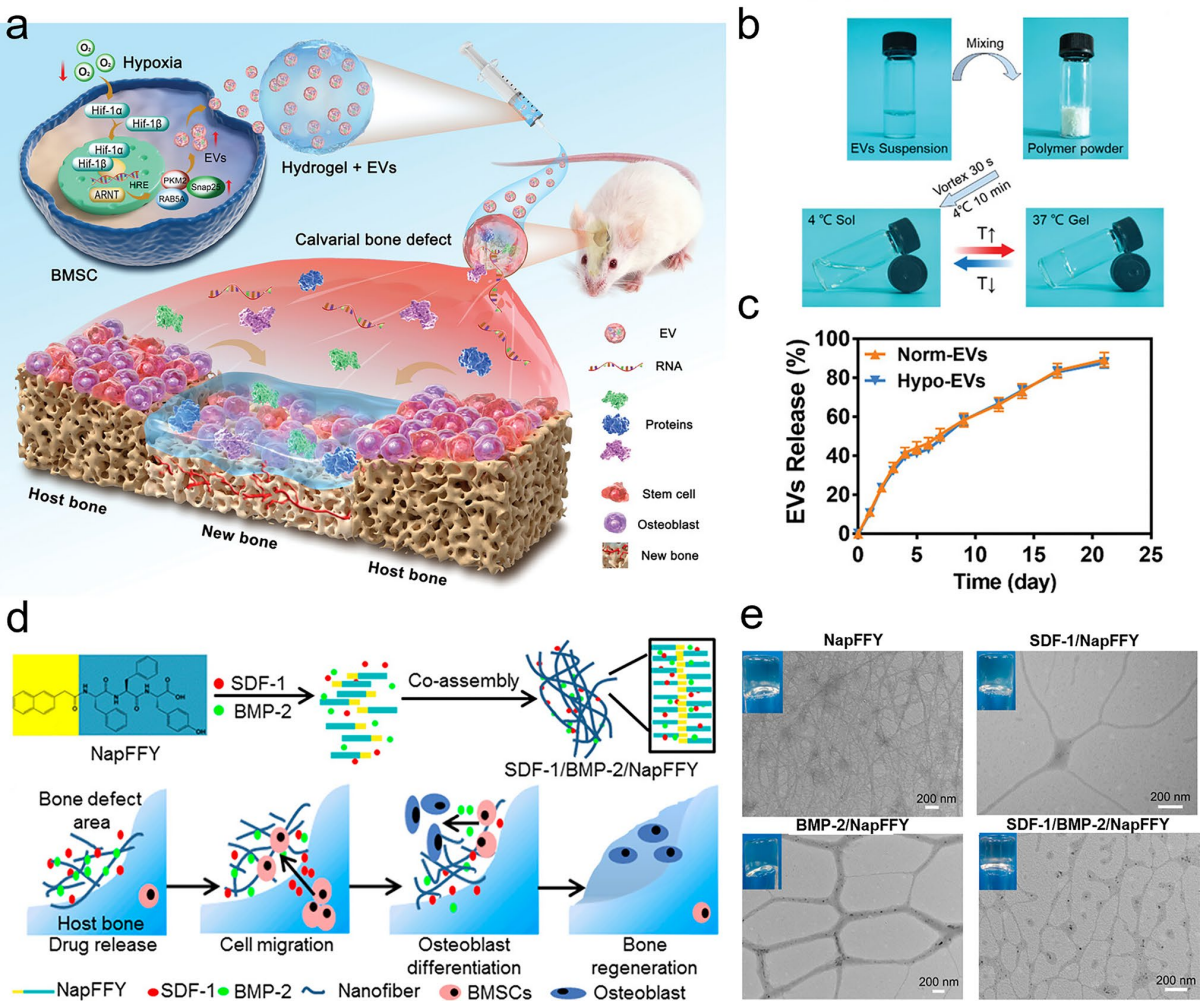
**a** Schematic illustration of the Hypo-EVs-loaded injectable PEG/polypeptide hydrogel for cranial defects regeneration. **b** PEG/polypeptide hydrogel exhibited rapid gelation at 37 °C. **c** Release curves of the EVs from the PEG/polypeptide hydrogel. **a**–**c** Reproduced with permission. Copyright 2023, John Wiley and Sons [16]. **d** Fabrication of an SDF-1/BMP-2 encapsulated NapFFY hydrogel for periodontal bone repair. **e** Distributions of SDF-1 and BMP-2 within the NapFFY hydrogel network. **d**, **e** Reproduced with permission. Copyright 2019, American Chemical Society [47]

Periodontal diseases, which involve damage to the tooth-supporting structures, can lead to tooth loss if left untreated. Regenerative periodontal therapies aim to arrest inflammation and heal damaged tissues, posing a significant challenge for the aging population. In addition, Infection is one of the causes of periodontitis, which is difficult to cure, and can often recur. Moreover, high oxidative stress exacerbates local inflammation and hinders the osteogenesis in such lesions. This section summarizes the bioactive molecules that are currently used for periodontal bone regeneration, including GFs, and bioactive ions. In one example, Liu et al. [196] prepared a thermosensitive polyethylene glycol diacrylate hydrogel (PEGD@SDF-1) loaded with SDF-1 and dithiothreitol (DTT) to promote periodontal bone regeneration. In both in vitro and rat models of periodontitis, the sustained release of DTT and SDF-1 led to the hydrogel with a significant reduction in ROS levels, reactivation of the Wnt/*β*-catenin signaling pathway in osteoblasts, and restoration of their osteogenic capability, thereby suggesting that a combination of DTT and SDF-1 could be promising for use in periodontal therapy. Similarly, Tan et al. [47] developed a Nap-Phe-Phe-Tyr-OH supramolecular hydrogel loaded with SDF-1 and BMP-2 (SDF-1/BMP-2/NapFFY) (Fig. 6d, e). In vitro and in vivo results demonstrated that the synchronized and continuous release of SDF-1 and BMP-2 from the hydrogel effectively promoted periodontal bone tissue regeneration. Importantly, the treatment of critically sized maxillary periodontal bone defects in rats with the SDF-1/BMP-2/NapFFY hydrogel achieved a superior bone regeneration rate of 56.7% (bone volume fraction) 8 weeks after implantation, suggesting its potential as a replacement for bone grafting in clinical settings related to osteogenic orthodontics. For the treatment of periodontal disease, the antibacterial and osteogenic activities of materials are particularly important. In this context, Wu et al. [94] prepared a hydrogel membrane for periodontal repair by incorporating ZnO NPs into chitin chains. The Zn^2+^ ions that sustainably released from the hydrogels not only inhibited bacterial growth, but also enhanced bone regeneration by accelerating cell differentiation and biomineralization.

**Fig. 7:**
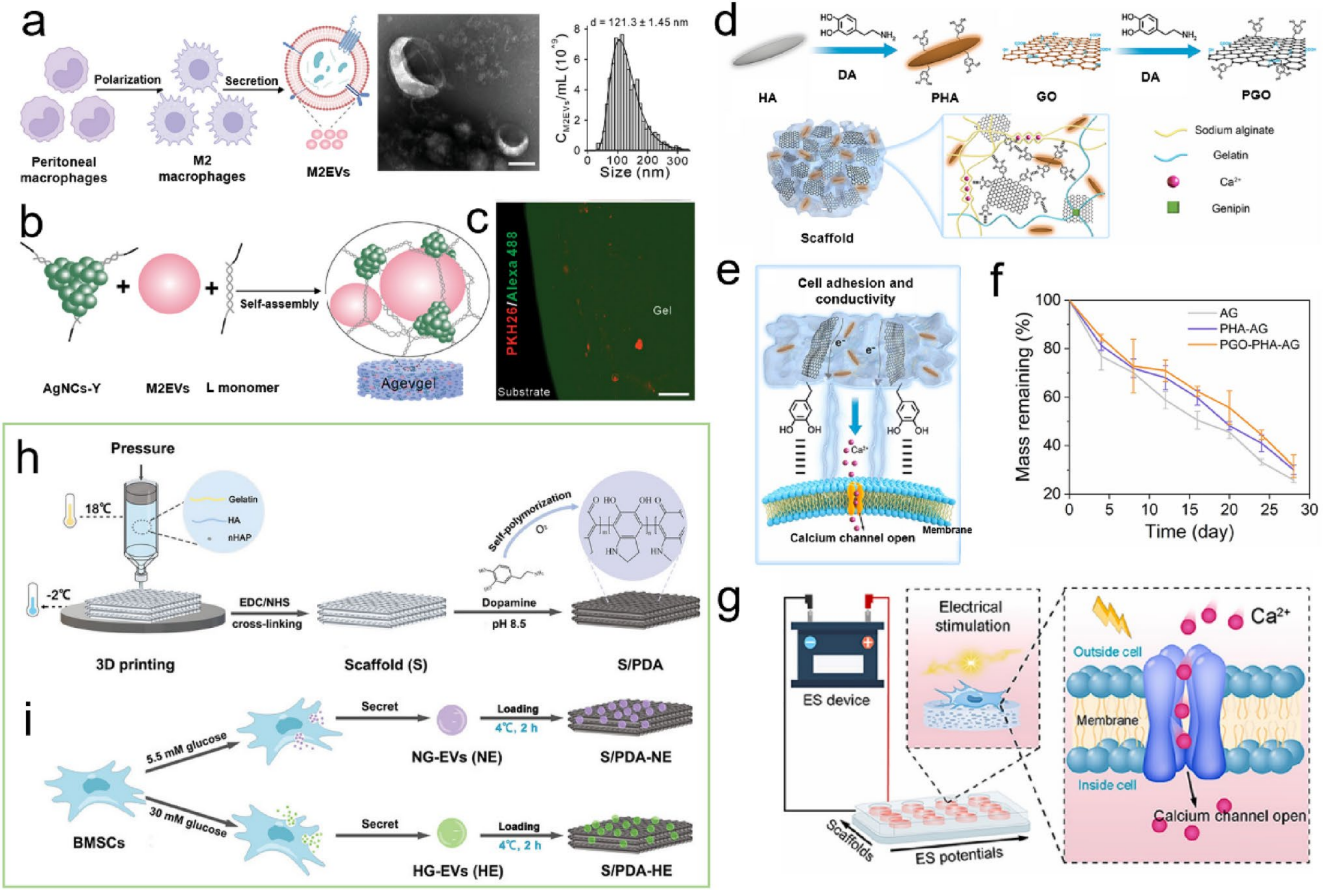
**a** Flow chart, micromorphology, and particle size of the M2EVs. **b** Schematic illustration of the M2EVs encapsulated during the Agevgel fabrication process. **c** Distribution of M2EVs in the Agevgel network. The M2EVs and AgNCs were stained with PKH26 and Alexa Flour, respectively. **a**–**c** Reproduced with permission. Copyright 2023, John Wiley and Sons [129]. **d** Schematic representation showing synthesis of the PGO-PHAp-AG scaffold. **e** Release of Ca^2+^ from the PGO-PHAp-AG scaffold. **f** Degradation curves of the different scaffolds in PBS solutions with type II collagenase. **g** High throughput electrical stimuli to enhance Ca^2+^ release from the scaffold into the cell to promote osteogenesis. **d**–**g** Reproduced under terms of the CC-BY license. Copyright 2022, Elsevier [93]. **h** Synthesis of the 3D-printed Gel/HA/nHAp scaffolds and **i** the EVs loading process. **h**, **i** Reproduced with permission. Copyright 2023, John Wiley and Sons [17]

Diabetic bone defect areas under diabetic conditions are known to exhibit inflammatory properties and excess ROS levels because of hyperglycemia, thereby leading to challenges in their repair. Owing to their reusability and biodegradability, DNA hydrogels have great potential as platforms for delivering bioactive molecules to treat periodontal disease. For example, Peng et al. [129] encapsulated silver nanoclusters (AgNCs) and M2 macrophage-derived EVs (M2EVs) into a DNA hydrogel (Agevgel) for diabetic alveolar bone repair (Fig. 7a–c). In this system, the AgNCs immobilized on the hydrogel exhibited a good antibacterial activity, while the M2EVs demonstrated anti-inflammatory and osteogenic capabilities. Moreover, in vitro results implied that the hydrogel prolonged the retention time of M2EVs compared to the use of M2EVs solutions or dispersions, with gradual M2EVs release being achieved over 7 days. Similarly, Li et al. [197] developed a biodegradable, anti-inflammatory, and osteogenic DNA hydrogel bioscaffold (ILGel) to gradually deliver inerleukin-10 (IL-10) for diabetic alveolar bone repair. The porous ILGel ensured that sustained IL-10 release could be achieved over 7 days to attenuate inflammation and induce osteogenesis. Furthermore, animal tests demonstrated that the ILGel significantly improved alveolar bone repair rate (93.42 ± 4.6%) on day 21 compared with that of pure IL-10-treated groups (63.30 ± 7.39%). Inspired by the catechol-chemistry of mussels, Li et al. [93] developed an SA/Gel (AG) hydrogel scaffold that incorporated PDA-functionalized GO (PGO) and HAp NPs (PHAp) to realize periodontal bone remodeling by regulating the inflammatory microenvironment at the lesion (Fig. 7d). PHAp endowed the scaffold with immunomodulatory ability because of PDA mediation and provided a suitable microenvironment for the bone remodeling process. Subsequently, the conductive scaffold induced by PGO facilitated bone regeneration by activating Ca^2+^ channels (Fig. 7e, g); thus, the Ca^2+^ released from the PGO-PHAp-AG scaffold (Fig. 7f) could enter the inner cells and stimulate the osteogenic signaling pathway to enhance osteogenic differentiation and periodontal bone reconstruction.

#### Osteochondral/Cartilage Repair

In contrast to bone defects repair in various disease models, the repair of osteochondral defects involves in the breakdown of articular cartilage and bellowing bone. Consequently, single-layer hydrogels have difficulty in restoring the functions of osteochondral cartilage tissues. To address this, Gan et al. [198] developed a bilayer hydrogel for repairing osteochondral defects in rabbit knee joints. Using a GelMA-polydopamine (GelMA-PDA) hydrogel matrix, PDA was found to trigger the in situ mineralization of HAp in the lower layer, and BMP-2 was incorporated into this layer to mimic the subchondral bone tissues. Subsequently, TGF-*β*3 was introduced into the upper layer to boost cartilage regeneration. Notably, the release rate of the mineralized HAp reached 65% by day 22. Owing to the large number of amines and thiol groups presented in its structure, the PDA components within the hydrogel were able to immobilize proteins or GFs via covalent and non-covalent bonds. Therefore, the sustained release of BMP-2 was achieved from the hydrogel, reaching a delivery rate of 50% on day 22. In this rabbit osteochondral knee joint defect model, treatment with the TGF-*β*3/BMP-2 bilayer hydrogel led to the generation of a smooth cartilage surface and repaired subchondral bone characteristics, thereby demonstrating that this bilayer hydrogel possessed a good osteochondral repair capability. Similarly, Fang et al. [199] developed a CS/gel-based hydrogel with a bilayer structure to restore damaged cartilage and subchondral tissue. In this bilayer hydrogel, in situ nHAp mineralization in the lower layer allowed direct interactions between the hydrogel and the subchondral bone, simultaneously endowing the lower hydrogel with a compressive strength of 2.5 MPa and a compressive strain of 40%. In addition, basic FGF (bFGF) was introduced into the upper layer to boost cartilage regeneration. Both in vitro and in vivo studies demonstrated that this bilayer hydrogel exhibited chondrogenic and osteogenic potential. Strategies based on free drugs and GFs have also been applied to repair osteochondral defects, and an economical and feasible therapeutic strategy has been developed. More specifically, Xing et al. [200] reported a gellan-gum (GG)-based hydrogel with a bilayer structure, which was formed through Ca^2+^-mediated secondary crosslinking. SA and HAp were then incorporated into the chondral and subchondral layers, respectively. In this bilayer network, the hydrogel acted as container to continuously release Ca^2+^, further facilitating neovascularization after 4 weeks of implantation, at the same time, both SA and GG could supply nutrients to support chondrogenic differentiation, finally achieving critical-sized osteochondral defect regeneration in rabbits after 8 weeks of therapy.

Cartilage plays a crucial role in determining the structures and functions of joints, exhibiting load-bearing and lubricating characteristics [201]. Cartilage is affected by trauma, long-term over-loading, and osteoarthritis; however, cartilage defects do not readily undergo self-repair due to a lack of blood vessels and neural tissues [202]. In an attempt to address this issue, Han et al. [203] used a GelMA-*co*-PAAm biohybrid hydrogel to encapsulate TGF-*β*2 for defective cartilage repair. The sustained release of TGF-*β*2 from this hydrogel enhance glycosaminoglycan (GAG) production in vitro, and further accelerated cartilage regeneration in vivo. According to the International Cartilage Repair Society (ICRS) histological grading, the TGF-*β*2-loaded biohybrid hydrogel groups (10.52 ± 1) possessed higher scores than the biohybrid hydrogel groups (8.67 ± 0.58). In addition, to obtain hydrogels with an adequate degradation rate for cartilage repair, a natural polymer derivative of GelMA was used as the hydrogel matrix, and oligomers of dopamine methacrylate (ODMA) were used to modulate the mechanical properties of the GelMA hydrogel. The obtained ODMA-GelMA hydrogel exhibited tough and resilient characteristics, which were attributed to the intercalation of ODMA. In addition, ODMA was able to immobilize bioactive molecules, thereby conferring good cell attachment and tissue integration abilities to the hydrogels. Indeed, in vivo animal experiments demonstrated that chondroitin sulfate and TGF-*β*3 delivery from this hydrogel promoted cartilage repair [204]. Furthermore, cartilage injuries are known to commonly trigger inflammatory reactions around the defects, which can interfere with the healing process and hinder tissue regeneration. Previous studies have demonstrated that catechol groups can sufficiently reduce the inflammatory response by regulating the polarization of macrophages [205–207]. With this in mind, Gan et al. [208] designed a collagen scaffold (Col) incorporated a PDA-modified HA complex (PDA/HA) to mimic the native cartilage ECM. In this scaffold, PDA/HA promoted cell adhesion and clustering, while also regulating the osteogenic behavior of BMSCs through its immunomodulatory capability. The Col/PDA/HA scaffold not only facilitated the polarization of M2 macrophages to create a conducive microenvironment for cartilage regeneration, but it also served as a hydrogel carrier for the sustained release of TGF-*β*3. Notably, overexpressed ROS and NO levels at the lesion site also trigger inflammatory reactions. Thus, inspired by natural cartilage with its abundant anionic molecules (e.g., hyaluronate, sulfonated GAG, and lubricin), Yu et al. [209] fabricated a carboxylate/sulfonate polyanionic hydrogel via Fe^3+^ crosslinking (CS-Fe hydrogel). This hydrogel exhibited a good mechanical adaptability and a high shear resistance. The valence changes between Fe^3+^ and Fe^2+^ via the Fenton reaction facilitated H_2_O_2_ elimination to generate ROS and oxygen; subsequently, the excess H_2_O_2_ and ROS reduced the level of NO via redox reactions and relieved inflammation at the lesion sites. This polyanionic hydrogel was therefore able to prevent the aggressive inflammation of chondrocytes/fibroblasts, ultimately facilitating cartilage regeneration. Creating a regenerative microenvironment can repair the impaired cartilage via relieving the chondrocyte senescence during the osteoarthritis development. Based on this, Zhu et al. [210] proposed an injectable hydrogel by integrating aging-related miR-29b-5p into bone marrow-homing peptide motif (SKPPGTSS)-functionalized self-assembling peptides. The combination of sustained release of miR-29b-5p from hydrogel, recruitment of endogenous synovial stem cell, and then differentiation to chondrocytes enabled chondrocyte rejuvenation and cartilage defects healing.

### Emerging Delivery Systems

#### Nano-/Microcarriers for Delivery

Conventional release systems frequently exhibit sudden drug release, which can lead to drug toxicity, and result in both economic and therapeutic inefficiencies. To address this, nanomaterials can be utilized as carriers for bioactive components within hydrogel networks. These materials, including inorganic/organic nanoparticles, MOFs, and NSs, have been integrated into hydrogel delivery systems to enhance the release profiles of bioactive components, thereby meeting the requirements of the bone repair process. In addition, microspheres sever as efficient carriers for hydrophobic molecules, thereby augmenting their use in drug delivery systems. Advancements in biotechnology have therefore led to the development of natural carriers, such as cell-derived exosomes, which hold promise for delivering various therapeutic molecules with an enhanced precision and efficacy. These innovative approaches offer new avenues for addressing challenges associated with conventional drug delivery systems, ultimately improving patient outcomes and treatment efficacies.

##### Nanomaterials

**Fig. 8:**
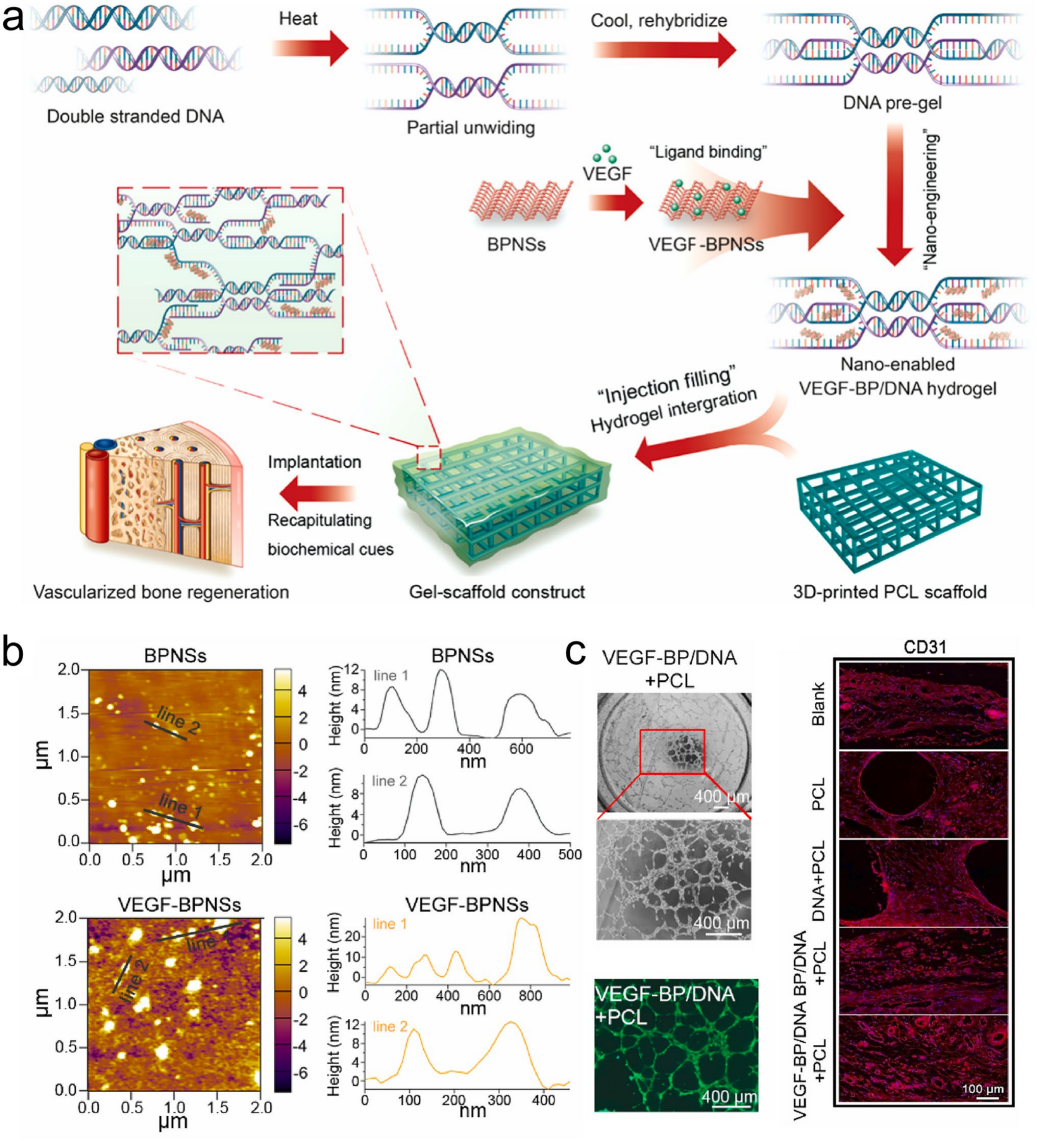
**a** Schematic illustration of the fabrication of a DNA hydrogel for vascularized bone formation. **b** AFM images of the BPNSs and VEGF-BPNSs. **c** Bright-field optical, fluorescence, and CD31 immunofluorescence images of the tube formation of HUVECs, Calcium-AM-stained HUVECs, and hydrogel-treated cranial defects, respectively. Reproduced under terms of the CC-BY license. Copyright 2022, Elsevier [213]

**Fig. 9:**
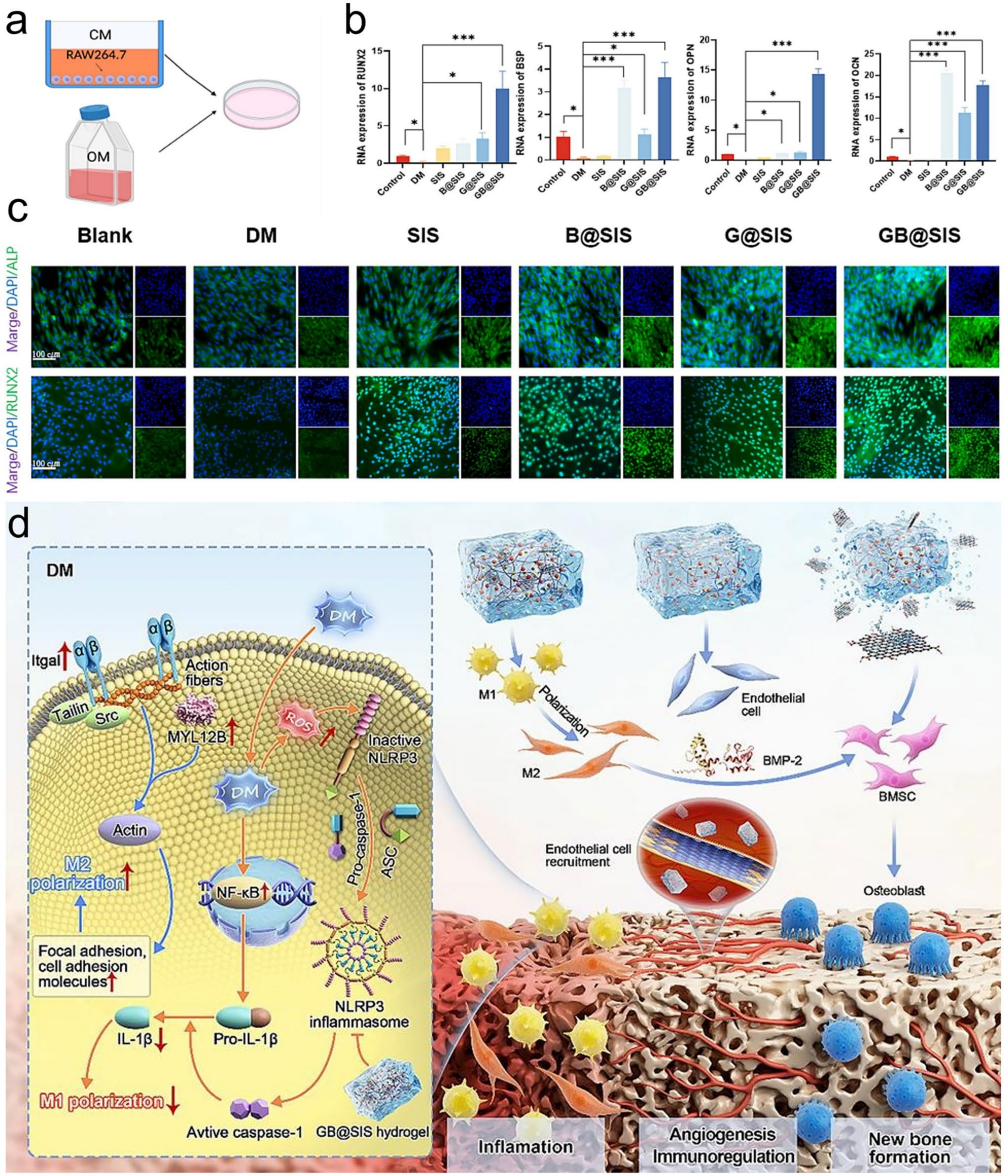
**a** Schematic illustration of the mixed medium containing macrophage-conditioned medium (CM) and osteogenesis-induced medium (OM). **b** Osteogenesis-related gene expression levels within the BMSCs cultured in an osteoimmuno-modulatory microenvironment for 14 days. **c** Immunofluorescence images of ALP and RUNX2 in the BMSCs cultured for 7 days. **d** Mechanisms of enhancing new bone formation induced by hydrogel delivery system. Reproduced with permission. Copyright 2024, Elsevier [23]

Nanomaterials have emerged as versatile platforms for drug delivery, offering precise control over the release kinetics and targeted delivery of therapeutic agents. Nanomaterials, which include nanoparticles and nanocapsules, possess unique physio-chemical properties that enable them to efficiently encapsulate drugs and protect them from degradation. These nanosized carriers can navigate biological barriers and accumulate at specific sites, thereby enhancing the drug bioavailability and minimizing off-target effects. MOFs have become a new generation of nanocarriers because of their high specific surface areas and porous structures, which help maintain the activities of various bioactive molecules. In this context, Lao et al. [25] prepared a Met-loaded zeolitic imidazolate framework (ZIF) nano-MOF incorporated into a GelMA hydrogel for diabetic bone regeneration. The synergistic effects of the released Met and Zn^2+^ suppressed inflammatory reactions by recovering mitochondrial functions, thereby strengthening the osteogenesis process, as demonstrated by both in vivo and in vitro experiments. Similarly, Qiao et al. [24] reported a nano-SIM-loaded ZIF-8 modified poly(ethylene glycol) diacrylate (PEGDA)/SA injectable biohydrogel (SIM@ZIF-8/PEGDA/SA, also called nSZPS), which was applied to stimulate osteogenic differentiation in hyperlipidemic conditions. Owing to the sustained release of Zn^2+^ and SIM, nSZPS presented excellent osteointegration and lipid-lowering abilities during bone repair in hyperlipidemic rats by reducing PPAR*γ* expression and increasing RUNX2 expression. In another study, Gong et al. [211] introduced PDA-mediated silk microfiber (PDA-mSF) and Met-loaded ZIF into SF/Gel hydrogel patches for periodontal hard and soft tissue regeneration via an immunomodulation strategy. In this system, PDA-mSF hindered the inflammatory reactions by inhibiting M1 polarization, while sustained release of Met from ZIF accelerated M2 polarization to secrete osteogenic cytokines. Simultaneously, Zn^2+^ was released from the decomposed ZIF system to promote bone regeneration. Therefore, all components of this delivery system created a favorable microenvironment for accelerating diabetic periodontal bone and soft tissue repair. More recently, 2D NSs, such as GO, BP, and MXene, have been reported as promising carriers for drug delivery applications owing to their large surface areas and unique surface chemical structures [212]. For example, BP NSs are biodegradable and conductive nanomaterials that exhibit a photothermal effect, and have been considered as ideal choices for drug delivery systems. Jing et al. [22] developed an Mg-modified BP-incorporated photosensitive and conductive GelMA hydrogel for the repair of infected skull defects. Based on the NIR-induced photothermal and photodynamic effects of BP, the hydrogel exhibited superior antibacterial and anti-inflammatory activities, thereby reducing the secondary damage caused by bacteria. Meanwhile, the BP NSs and bioactive Mg^2+^ released from the hydrogel contributed to neurite outgrowth and innerved bone remodeling. Similarly, Huang et al. [107] coated a TA-Mg^2+^ chelate layer on the surfaces of BP NSs to provide a methacrylate SF (SFMA) hydrogel (SFMA-BP@TA-Mg, denoted as SFBTM) that exhibited good osteo-immunomodulation performance and osteogenesis ability. In vivo results revealed that the SFBTM hydrogel significantly enhanced vascularized osteogenesis by modulating the immune microenvironment, which was attributed to the gradual release of BP and Mg^2+^. BP can also serve as a carrier for the delivery of GFs, proteins, and polysaccharides. In this context, Miao et al. [213] introduced VEGF-loaded BP into a DNA dynamic hydrogel for integration with a 3D-printed polycaprolactone (PCL) scaffold to build a bioactive gel scaffold for vascularized cranial defect repair (Fig. 8a). The BPNSs not only imparted positive effects on the mechanical strength of the DNA hydrogel, but they also endowed the gel scaffold with the ability to load and deliver GFs in a sustained manner (Fig. 8b). The gel scaffold enhanced vascularized bone regeneration owing to the synergistic effects of VEGF and BP, providing great potential for application in irregular bone defect repair (Fig. 8c). Other NSs, including GeP and GO, have also been frequently utilized as nanocarriers for the controlled release of various bioactive molecules. For example, Sheng et al. [23] fabricated a BMP-4-loaded GO composite porcine small intestinal submucosal (SIS) hydrogel (GB@SIS) for diabetic bone regeneration. The BMP-4 incorporated on the GO NPs was sustainably released from the hydrogel, and synergized with the SIS ECM to promote M2 macrophage polarization and secrete BMP-2, thereby enhancing bone regeneration. In addition, in vivo experiments carried out using critical-size skull animals with DM demonstrated that the GB@SIS hydrogel also exerted immunomodulatory effects by suppressing the NLRP3 signaling pathway (Fig. 9). In addition, Xu et al. [19] developed a Cu^2+^-loaded GeP hybridized GelMA hydrogel (GelMA/GeP@Cu) by UV photopolymerization. In this system, the Cu^2+^-modified GeP NSs not only improved the stability of BP, but also enabled the sustained release of Cu^2+^. Consequently, the GelMA/GeP@Cu hydrogel promoted angiogenesis and neurogenesis, ultimately enhancing cranial defect repair. It should be noted that the conductive GeP@Cu NSs showed biodegradability, unlike commonly used graphene and carbon nanotube materials, thereby demonstrating their potential for bone repair in clinical applications.

**Fig. 10:**
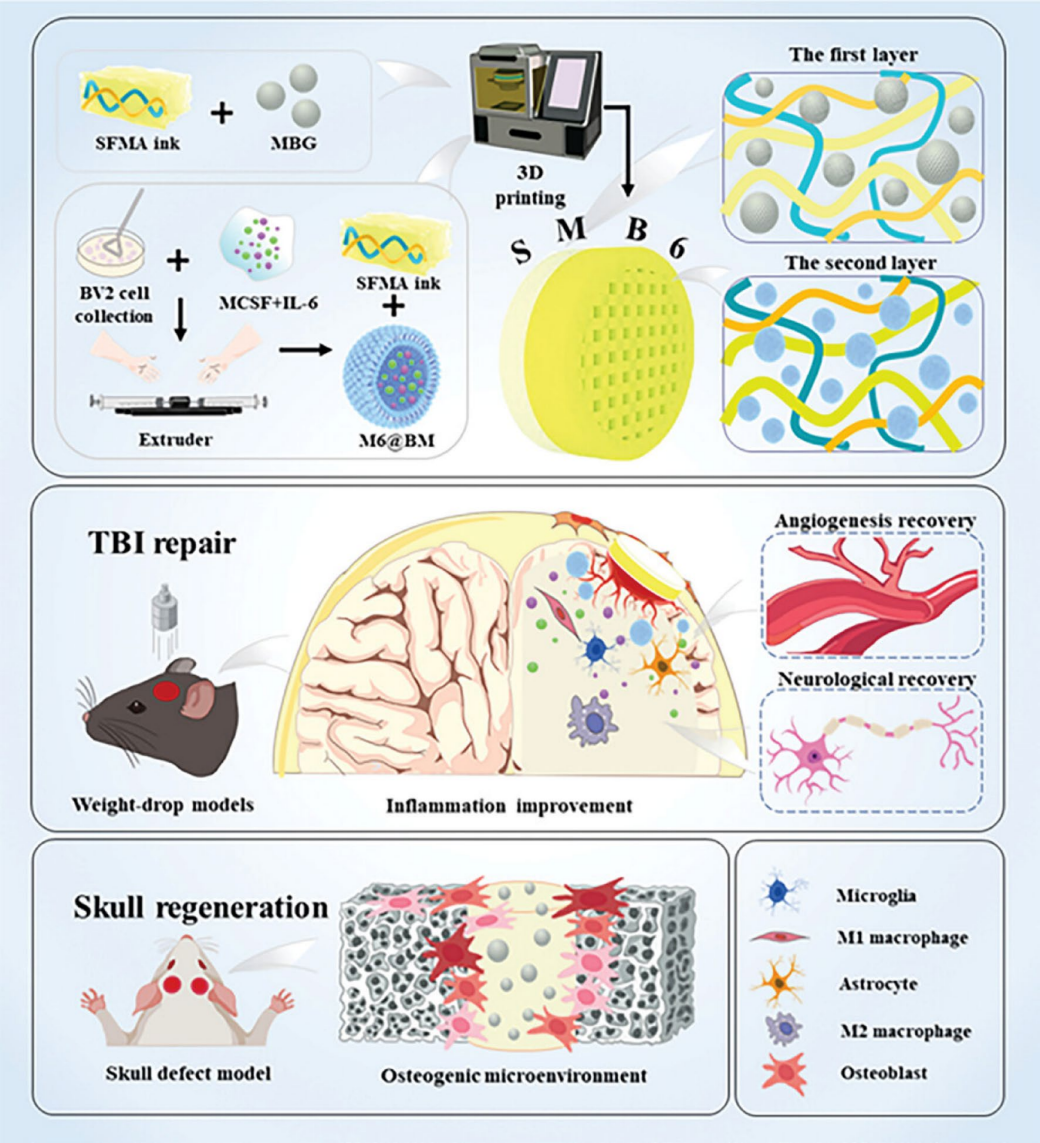
Schematic illustration of the simultaneous reparation of TBI and cranial defects using the biomimetic cranial-brain hydrogel patch. Reproduced with permission. Copyright 2024, John Wiley and Sons [216]

**Fig. 11:**
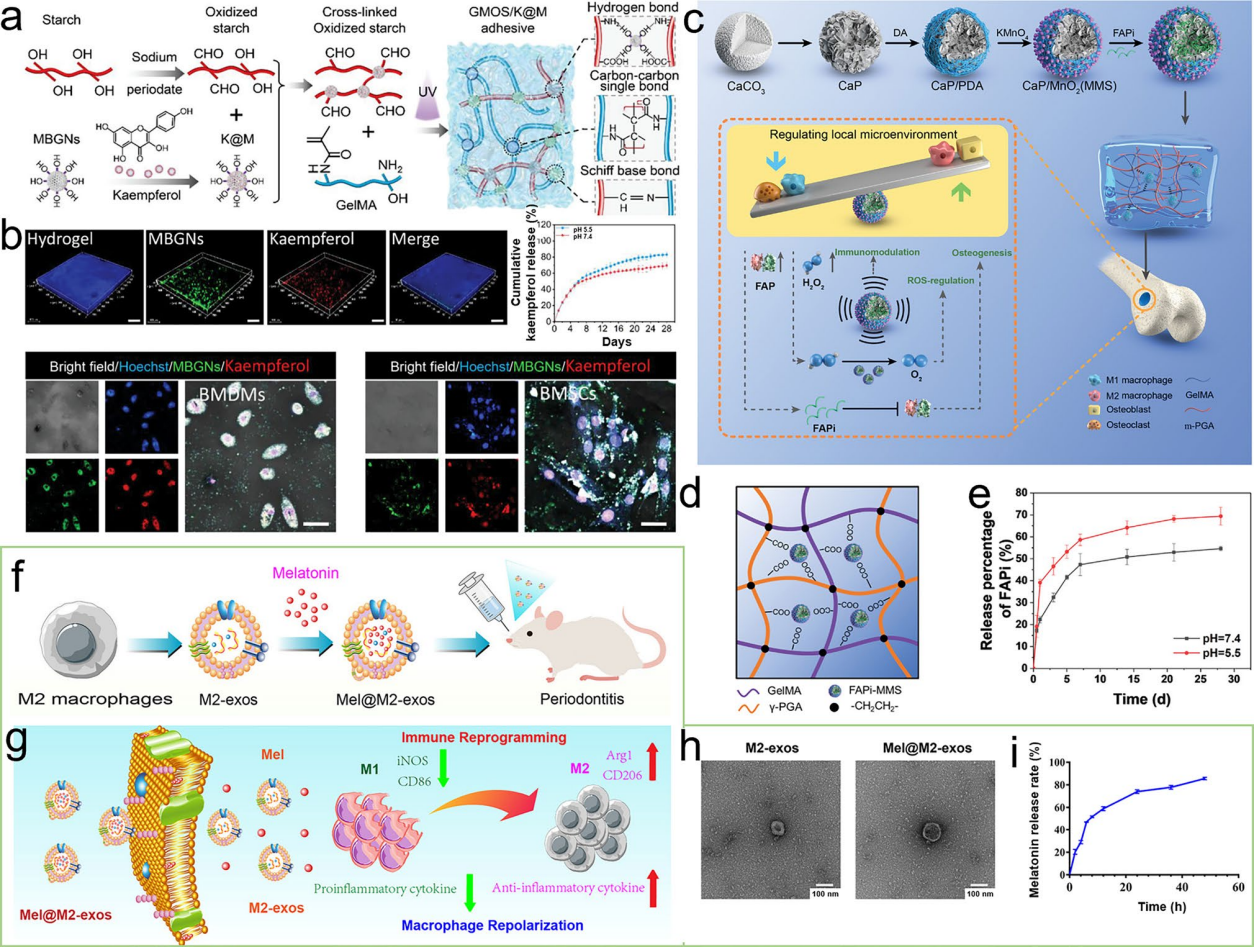
**a** Schematic illustration of the kaempferol-decorated GMOS/K@M hydrogels. **b** Kaempferol distribution pattern and release curve from the hydrogel network. **a**, **b** Reproduced with permission. Copyright 2023, John Wiley and Sons [20]. **c** Preparation of the FAPi-loaded MnO_2_-CaP microspheres composite hydrogels for osteoporotic bone healing. **d** Interactions between the FAPi-loaded microspheres and the hydrogel chains. **e** Cumulative release profiles of FAPi from the composite hydrogels. **c**–**e** Reproduced with permission. Copyright 2022, John Wiley and Sons [231]. **f** Fabrication of the Mel-engineered exosomes-incorporated GelMA hydrogel for periodontal bone defect therapy. **g** Schematic diagram of Mel delivery from the Exos to promote immunomodulation process. **h** SEM images of the Mel-engineered Exos. **i** Mel release curve. **f**–**i** Reproduced under terms of the CC-BY license. Copyright 2023, John Wiley and Sons [87]

Nanoparticles have demonstrated good potential for delivery system applications due to their ability to improve the stabilities and efficacies of drugs and bioactive molecules. In this context, Huang et al. [21] designed fluorinated PDA-coated HAp NPs (HAp@PDA-F NPs) incorporating quaternized and methacrylated CS (CS/HAp@PDA-F) hydrogels via a photo-crosslinking strategy. The CS/HAp@PDA-F hydrogel possessed a sustained oxygen-supplying capability, which significantly promoted the expression of osteogenesis-related genes under hypoxic conditions. In vivo experiments revealed that this hydrogel promoted a high bone volume and mineral density. Moreover, BG has been reported as a promising candidate for use to as multifunctional carrier of bioactive molecules to assist their delivery to target sites [214]. For example, Kaempferol-loaded mesoporous BG NPs (MBGNs) were incorporated into oxidized starch/GelMA to prepare an adhesive hydrogel (GMOS/K@M) via a Schiff-base reaction and free-radical polymerization (Fig. 11a). This adhesive assisted in osteoporotic fracture fixation and enhanced osteoporotic fracture healing. More specifically, the sustained release of Ca^2+^ and SiO_4_^4−^ ions from the adhesive facilitated osteogenesis via the PI3K/AKT signaling pathway, while kaempferol inhibited osteoclastogenesis and undermined bone resorption via the NF-κB signaling pathway, thereby boosting osteoporotic bone regeneration. In this delivery system, kaempferol was uniformly distributed within the hydrogel matrix and could be gradually released, achieving ≤ 80% release over 28 days under acidic conditions (Fig. 11b) [20]. Furthermore, Huang et al. [215] developed a tyramine-grafted poly(L-glutamic acid) composite hydrogel (PLG-g-TA) containing VEGF and Sr-doped borosilicate BGNPs (Sr-BGNPs) (i.e., denoted as PLG-g-TA/VEGF/Sr-BGNPs) via horseradish peroxidase-promoted crosslinking. Bioactive ions and VEGF were gradually released from the PLG-g-TA/VEGF/Sr-BGNPs hydrogel, and in vitro tests revealed that the hydrogel promoted the osteogenic differentiation of rat BMSCs by upregulating osteogenesis-related gene and protein expression, thereby enhancing tube formation and suppressing osteoclast bone resorption. After implantation into rat cranial defects, the hydrogel also promoted bone formation by increasing the osteogenic and angiogenic biomarker levels, providing a favorable strategy for large-scale bone defect repair. In addition to their application in the repair of common cranial defects, BGNPs can also be applied to treat traumatic brain injury (TBI)-induced cranial loss. More specifically, Zhou et al. [216] reported a 3D-printed cranial-brain patch (SMB6) with a bilayer structure consisting of a mesoporous BGNPs-integrated SFMA layer and a supporting SFMA hydrogel layer, which simultaneously achieved the repair of cranial defects and TBI (Fig. 10). This hydrogel inhibited brain edema, reconstructed blood vessels and nerves, and relieved inflammatory reactions. Si ions were sustainably released from the hydrogel, and the delivery rate was found to slow after 12 days, thereby enhancing deposition of the bone matrix and the recruitment of BMSCs. In addition, the porous BGNPs-loaded hydrogel layer exhibited a structure similar to that of natural bone tissue, which promoted the adhesion, proliferation, and differentiation of osteoblasts, eventually enhancing cranial defect repair. The second layer provided an ECM matrix for biomimetic microglial delivery, further repaired the severe microenvironment, and recovered the neuron and vascular function. Open fractures tend to exhibit high incidences of postoperative complications, and the fracture sites are difficult to self-heal due to an insufficient blood supply. In the clinical setting, the main therapeutic approach in the early period is debridement, which promotes the closure and fixation of open fracture [217]. However, the infection rates of open fractures can reach 27% even after complete debridement during the “golden period” of 6–8 h after injury [218, 219]. Thus, the development of an effective fixation and anti-infective biomaterial for open fracture repair is of particular importance. In this context, BGNPs can be used as carriers to deliver drugs and essential ions to achieve open fracture healing. For example, Yang et al. [220] designed a bone adhesive by incorporating vancomycin (VAN)-loaded MBGNs into Gel/OS hydrogel via covalent crosslinking through Schiff-base reactions. In vitro results revealed that the hydrogel adhesive promoted BMSCs proliferation, upregulated the expression of ALP, RUNX2, and OPN, and boosted calcium nodule deposition, as evidenced by a skull fracture model. In this system, the hydrogel adhesive was firmly and stably attached to the fracture fragments, and VAN was released from the MBGNs to suppress bacterial growth. Additionally, the MBGNs were found to deliver bioactive ions for angiogenesis and osteogenesis. This concept therefore provides a favorable strategy for open fracture healing by combining antibacterial drugs with functional ions. In addition to the wide application of nanoscale BG, BG microparticles decorated with amino groups (NBG) have been incorporated into an SA/GG matrix to fabricate an electrostatically reinforced hydrogel (CAG) for cranial defect repair. Compared with traditional BG, NBG possessed a uniform porous structure and a high compressive strength of 66 kPa. Incorporation of the NBG slowed down the hydrogel degradation rate (~ 54% on day 28) to match the bone regeneration, thereby achieving an optimal osteogenesis efficacy. Following degradation of the hydrogel network, bioactive ions such as Ca and Si, were released from the hydrogel over periods of 28 and 14 days, respectively, thereby enhancing HAp biomineralization to assist bone formation in vivo. Moreover, the CAG hydrogel promoted M2 polarization and upregulated osteogenic gene expression to accelerate new bone formation. The incorporating of functionalized BG into biomaterials may therefore represent a simple method for improving the interfacial compatibility between bone tissue and biomaterials [221]. Dual release of different NPs from hydrogel has been developed in recent years. Kang et al. [222] integrated kartogenin-loaded PDA (KGN@PDA) NPs and miRNA-loaded CaP (miRNA@CaP) NPs into 2-ureido-4(1H)-pyrimidinone functionalized gelation hydrogel film for osteochondral defect repair. The hydrogel achieved sustained release of KGN@PDA and miR-26a@CaP, to enhance MSCs differentiation to chondrogenesis and osteogenesis through the JNK/RUNX1 and GSK-3*β*/*β*-catenin pathway, respectively.

In addition, biodegradable polymers, including heparin [223], CS [224, 225], Gel [110], SA [226, 227], albumin [228], and HA [229, 230], have been considered as essential choices for encapsulating and delivering bioactive molecules. For example, Wu et al. [223] fabricated a BMP-2-loaded PDA/heparin NPs (BPDAH) functionalized Gel/PEGDA/2-(dimethylamino)ethyl methacrylate (GPEGD) hydrogel (BPDAH-GPEGD) for mandibular bone regeneration. BPDAH-GPEGD achieved BMP-2 sustained release (< 50%) over 30 days, which was superior to the release behavior of BMP-2 from the GPEGD hydrogel alone (i.e., > 80%) within 6 days, thereby suggesting that the PDAH possessed a good ability for BMP-2 loading and delivery. At times of 4 and 8 weeks after implantation, the hydrogel-treated mandibular bone defects presented optimal bone volumes and highly efficient bone regeneration characteristics, as indicated by the micro-CT and histology results. Furthermore, proteins can also act as carriers for the delivery of GFs. Mimicking by natural bone-like ECM, Gan et al. [228] developed an Arg-Gly-Asp (RGD)-grafted CS/biphasic CaP scaffold by the addition of BMP-2-loaded BSA NPs. Both in vitro and in vivo experiments indicated that the delivery of RGD and BMP-2 synergistically promoted cell attachment, spreading, and osteogenic differentiation, successfully creating a mild microenvironment for femoral defect regeneration. Nucleic acids-based nanomaterials can also be used to load biological molecules. Sun et al. [145] introduced miR29c-loaded tetrahedral framework nucleic acids into GelMA scaffold, achieved a sustained release of miR29c to create a growth environment for BMSCs, finally enhancing cranial defects repair. Based on the synthetic polymers, Wang et al. [149] designed a hybrid copolymers deblock NPs/PEG-b-poly(lactide)-b-dimethacrylate (PEG-b-PLA-b-DM) hydrogel to sustained release Wwp1 siRNA-loaded NP for 28 days during femur fracture healing. The NP was fabricated by the copolymerization of dimethylaminoethyl methacrylate, propylacrylic acid, and butyl methacrylate (pDMAEMA-b-p(DMAEMA-co-PAA-co-BMA)). The hydrogel-mediated delivery could prolong the knockdown of Wwp1 siRNA compared to Wwp1 siRNA-loaded NPs alone, showing significantly enhanced new bone formation and accelerated defects healing.

##### Microspheres

Microspheres, which can be composed of inorganic materials, natural polymers, and synthetic polymers, are widely used as drug delivery system to overcome the shortcomings of bioactive molecules. These microspheres are typically biocompatible, offer a high bioavailability, and enable sustained drug release over extend periods. More specifically, microspheres can encapsulate various types of bioactive molecules, such as small molecules, proteins, and nucleic acids, and can be conveniently administered via a syringe needle. Due to the fact that the natural bone matrix is rich in calcium, Ca^2+^ -based microspheres have been widely used to enhance new bone formation. In addition, Li et al. [83] integrated Res and Dex into a poly(D,L-lactide)-PEG-poly(D,L-lactide) (PLEL) hydrogel to create a mild microenvironment. Based on the synergistic effects of sustained Res and Dex release from the porous carbonate HAp microspheres, inflammatory reactions were hindered and stem cell differentiation was promoted to enhance osteoporotic bone defect regeneration. Similarly, Chen et al. [231] incorporated fibroblast-activating protein inhibitor (FAPi)-loaded MnO_2_-coated CaP microspheres (FAPi-MMS) into a methacrylated poly(glutamic acid)/GelMA (m-PGA/GelMA) hydrogel (Fig. 11c, d), wherein MnO_2_ was employed to reduce H_2_O_2_ levels and promote oxygen production. FAPi released from the hydrogel reached ~ 70% within 28 days under acidic conditions (pH 5.5) (Fig. 11e), thereby regulating the immune response and facilitating new bone formation. In addition, Gong et al. [232] integrated BMP-2-loaded CaCO_3_ microspheres into a fibrin-glue hydrogel (FC-B) for application in tibial defect repair. Owing to its be decoration with casein and heparin, BMP-2 demonstrated a high affinity for CaCO_3_ microspheres, which led to the sustained release of BMP-2 from the microspheres over 21 days. Notably, 8 weeks after implantation, the hydrogel-treated bone defects were almost healed. Hydrogel microspheres have also been reported as promising carriers for bioactive molecule delivery because of their minimally invasive characteristics. Using microfluidic technology, Wu et al. [15] fabricated a BMSCs-loaded porous GelMA hydrogel microsphere with a uniform particle size (300 μm) and pore size (50 μm). BMSCs emerged on the microsphere surface and penetrated the pores, with sustainably release being achieved at the lesion upon degradation of the hydrogel network; in vitro tests suggested that the cell release process could be maintained over 8 days. Importantly, these hydrogel microspheres exhibited great osteogenic potential both for in vitro and in vivo osteoporotic bone regeneration. Similarly, hydrogel microspheres can also effectively deliver metal ions. Inspired by magnets to capture metals, Zhao et al. [110] reported a bisphosphonate-grafted GelMA hydrogel microsphere that captured Mg^2+^ (GelMA-BP-Mg), thereby enabling the sustained release of Mg^2+^. In this delivery system, the GelMA-BP-Mg hydrogel microspheres could capture 0.6 at% Mg^2+^, achieving sustained release over 18 days. Moreover, Robert et al. [233] embedded Dex-loaded PLGA microspheres into an acellular agarose hydrogel for use in osteochondral repair. They found that the sustained release of Dex could be prolonged for at least 99 days. Notably, numerous commercial products, including Lupron Depot^®^ and Nutropin Depot^®^, are based on polymer microspheres [234, 235].

##### Engineered Extracellular Vesicles

EVs are heterogeneous nanometer-sized particles that can be released by various cells, and which act as protective carriers for proteins, lipids, and DNA fragments [236, 237]. It has been demonstrated that engineered EVs can be produced, which integrate various functional components to achieve an optimal therapeutic efficacy. Their biocompatibility, low immunogenicity, and ability to cross biological barriers make them ideal candidates for advanced therapies. Ongoing research aims to optimize EVs production, loading methods, and targeting strategies to maximize their effectiveness in clinical applications. In summary, engineered EVs are a novel and versatile tool in regenerative medicine, offering targeted, efficient, and less invasive solutions for bone healing and repair. For example, Sun et al. [238] engineered Exos with abundant BMP-2 levels and introduced them into a GelMA hydrogel network, these Exos were collected from mouse embryonic fibroblasts (NIH-3T3) via gene manipulation and ultracentrifugation techniques. In vitro and in vivo tests demonstrated that this hydrogel could effectively enhance the osteogenic differentiation of BMSCs and improve in situ cranial formation. Combination therapeutics based on EVs and mRNA have also been developed. For example, Kuang et al. [239] integrated BMSCs-derived Exos/miRNA-26a (Exo@miR-26a) into a hydrogel, which was subsequently implanted at the injured sites. As a result, bone regeneration was significantly promoted due to the local release of Exo@miR-26a, which could be ascribed to the angiogenic effects of the Exos and the osteogenic effects of miR-26a. It was also suggested that the Exos from the BMSCs could inhibit osteoclast differentiation-related gene expression to realize bone repair, instead of causing osteoclast damage. In addition, based on the immunomodulatory function of M2 macrophage-derived Exos (M2-Exos), Cui et al. [87] encapsulated Mel and introduced the resulting Mel@M2-Exos components into GelMA hydrogels (Fig. 11f). This hydrogel constantly released Mel@M2-Exos to accelerate periodontal bone regeneration, then subsequently released M2-Exos to alleviate chronic inflammatory reactions and guide new bone formation processes (Fig. 11g–i). Furthermore, liposome-like ethosomes (Eth) exhibit high deformability and high encapsulation rate characteristics, leading to their application in the delivery of hydrophilic and hydrophobic drugs. In this context, Li et al. [240] developed a GelMA/GG methacrylate (Eth-DFO@GelMA/GGMA) scaffold containing DFO-loaded Eth via photo- and ion-mediated crosslinking. DFO was sustainably released from the Eth-DFO@GelMA/GGMA scaffold, significantly promoting endothelial cell migration, tube production, ALP expression, and mineralized matrix deposition in osteoblasts. Rat cranial defect experiments revealed that this scaffold facilitated angiogenesis and bone regeneration by activating the HIF-1*α* signaling pathway. As a cell-free therapeutic technique, EVs are effective in promoting bone regeneration, osteogenesis, biomineralization, and vascularization, thereby drawing great attention in the context of bone regeneration [241]. However, few clinical trials have been carried out into the application of EVs for this purpose, potentially due to the lack of an efficient strategy for their simultaneous enrichment and purification.

#### Smart Delivery

Smart hydrogel delivery systems are powerful tools for use in bone tissue engineering. These systems utilize microenvironmental triggers, such as temperature, pH, and redox compositions, to achieve passive tuning. In addition, external stimuli, such as US and NIR irradiation, enable the on-demand release of bioactive molecules from the hydrogels. This smart approach allows for precise control, preventing burst or off-target release to minimize potential side effects. The following sections review the various delivery hydrogels fabricated over the past ten years.

##### Photothermal-Responsive Delivery

**Fig. 12:**
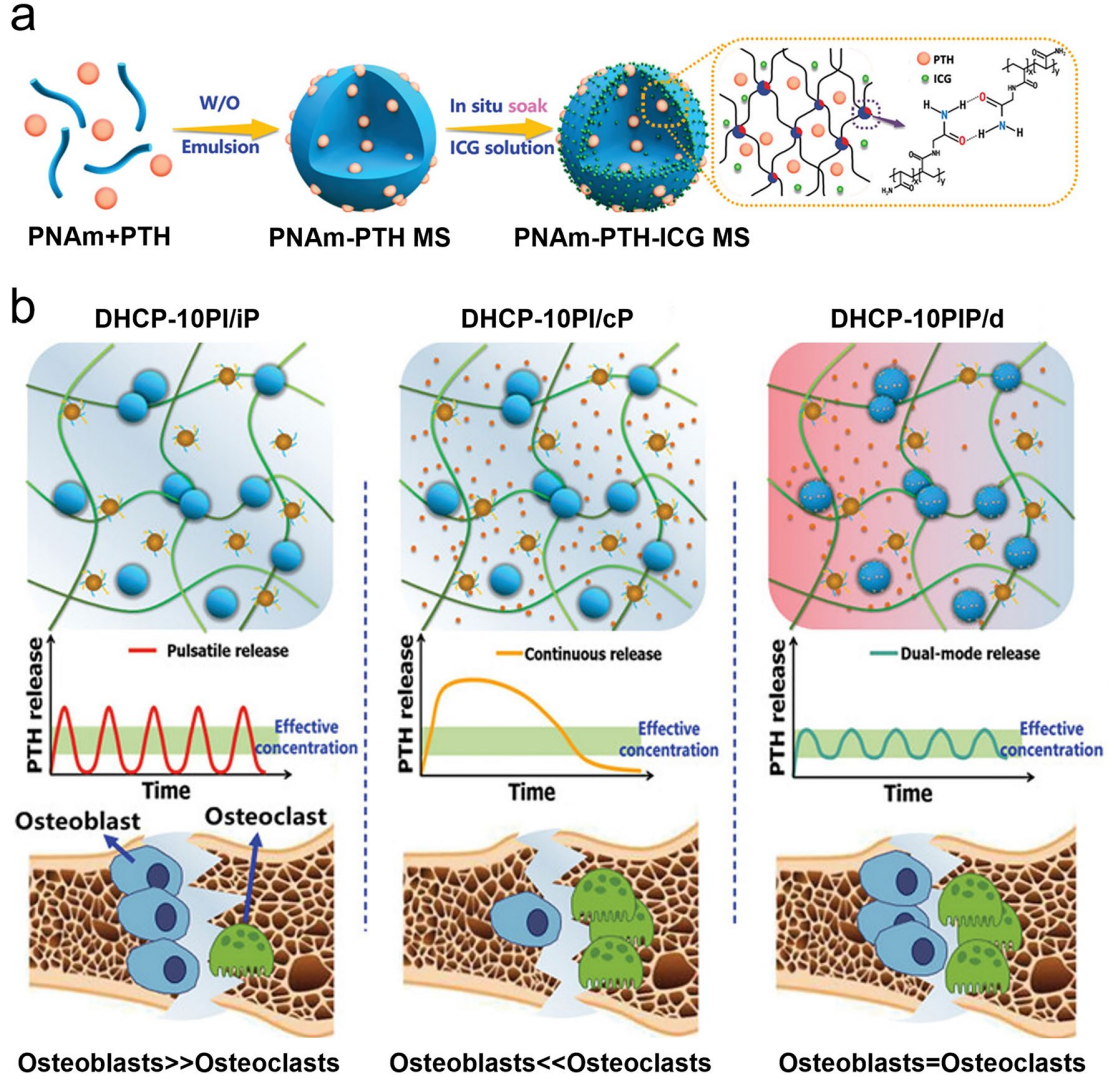
**a** Schematic illustration of the fabrication of the NIR-responsive PIP MSs via a water/oil emulsion. **b** Activities of the osteoblasts and osteoclasts under different release behaviors. Reproduced with permission. Copyright 2021, John Wiley and Sons [29]

Photothermal hydrogel delivery systems, which convert light into heat, offer a method for remotely controlling the release of drug/bioactive molecules via external stimuli. For example, NIR light-induced photothermal hydrogels have been widely used in localized drug delivery systems, wherein hydrogel networks or other carriers undergo phase transitions to deliver bioactive molecules under NIR stimuli. In this context, Liu et al. [11] designed an NIR-activatable scaffold with a dual-mode PTHrP-2 release capability to synergistically enhance osteogenesis and angiogenesis for efficient bone regeneration. This scaffold incorporated PTHrP-2-loaded carbon dot-doped MBGNs into a thermosensitive poly(N-isopropylacrylamide-*co-*N-hydroxymethylacrylamide) hydrogel (denoted as CDBGN/P(NIPAM-*co*-NMA)). The resulting hydrogel achieved reversible phase transitions in response to NIR irradiation, allowing precise control of the on-demand pulsatile and long-term slow release of PTHrP-2. In vivo experiments demonstrated significant improvements in the repair of critical-sized femoral defects in rats, suggesting the potential for developing effective dual-mode delivery systems for bone regeneration. Microspheres can also be applied to achieve the pulsatile release of drugs via a sol-gel transition. More specifically, Kuang et al. [29] developed a PTH-encapsulated injectable hydrogel composed of in situ-generated CaP NPs (ICPN) coordinated with poly(dimethylaminoethyl methacrylate-*co*-2-hydroxyethyl methacrylate) (ICPN-DHCP). Photothermal-responsive microspheres (PIP MSs) were introduced into this multifunctional system, enabling dual-mode delivery with the sustained and pulsatile release of PTH under NIR irradiation. Importantly, this system facilitated the successful repair of ovariectomy rat cranial defects by simultaneously promoting the activities of osteoblasts and osteoclasts simultaneously (Fig. 12). Similarly, Wan et al. [242] introduced NIR-responsive PDA-functionalized magnesium calcium carbonate microspheres into a thermo-responsive hydroxybutyl chitosan hydrogel for the sequential release of aspirin and BMP-2. Aspirin was initially released to relieve inflammatory reactions and promote M2 polarization; subsequently, BMP-2 was released under NIR light irradiation to induce new bone formation, as evidenced by cranial defect experiments in Sprague–Dawley rats. Additionally, the photothermal effect of the NIR-responsive hydrogel is capable of promoting bone tissue regeneration by modulating cellular gene expression. By integrating inducible transgene expression and NIR-responsive hydrogel technologies, Sanchez-Casanova et al. [51] introduced genetically engineered MSCs and plasmonic gold NPs into fibrin hydrogels. Upon exposure to an NIR laser, the gold NPs in the hydrogel converted NIR light into heat, thereby prompting the release of BMP-2 from the cell structure. This controllable delivery of BMP-2 was shown to enhance the formation of mineralized tissue at the lesion site, as evidenced upon its application in cranial defect repair experiments. In addition to particle-like carriers, various NSs have also been demonstrated to confer hydrogel matrices with good photothermal property. For example, Zhao et al. [243, 244] reported a series of BP NSs composite hydrogel scaffolds for tumor inhibition, anti-infection, and promoted osteogenesis under mild hyperthermia microenvironments created by NIR-induced photothermal effects. Furthermore, based on the 3D printing technique, Zhang et al. proposed a Gel/bioceramic core–shell scaffold by incorporating doxorubicin (DOX) within Gel (core) and NIR-responsive SrCuSi_4_O_10_ (SC) NSs/*β*-tricalcium phosphate (*β*-TCP) (shell). The SC NSs conferred the scaffold with a good photothermal performance under NIR-II laser stimuli, further facilitating the sol–gel transition of the Gel component and triggering the on-demand release of DOX for cancer cell apoptosis. The sufficient hollow channels remaining in the scaffold supported the ingrowth of new bone tissue. Simultaneously, the bioactive ions released during the degradation of SC promoted vascularized bone reconstruction [245]. The majority of conductive nanomaterials also present NIR-absorbing photothermal characteristics that are useful for photothermal therapy applications at lesion sites, due to their rapid light-to-heat conversion capabilities. In this context, Chen et al. encapsulated Dex within an MXene composite, poly(N-isopropylacrylamide)-*co-*N-(hydroxymethyl) acrylamide (PNN), to develop an NIR-responsive hydrogel (D-MPNN hydrogel). Under NIR irradiation, MXene converted the NIR light into heat, and facilitated shrinkage of the temperature-sensitive PNN at 42 °C to promote the controllable release of Dex for cranial reconstruction [82]. Moreover, based on the electrodeposition technique, Wang et al. proposed an NIR-responsive reduced GO (rGO)-loaded CS hydrogel (CS/rGO) with encapsulated teriparatide for osteoporotic bone repair. Under NIR stimulation, the local delivery of teriparatide from the CS/rGO film was achieved using the photothermal conversion effects of rGO, which increased the hydrogel temperature and regulated the teriparatide delivery behavior. In vivo experiments showed that this hydrogel film enhanced bone regeneration and accelerated blood vessel reconstruction [246].

##### Ultrasonication-Responsive Delivery

**Fig. 13:**
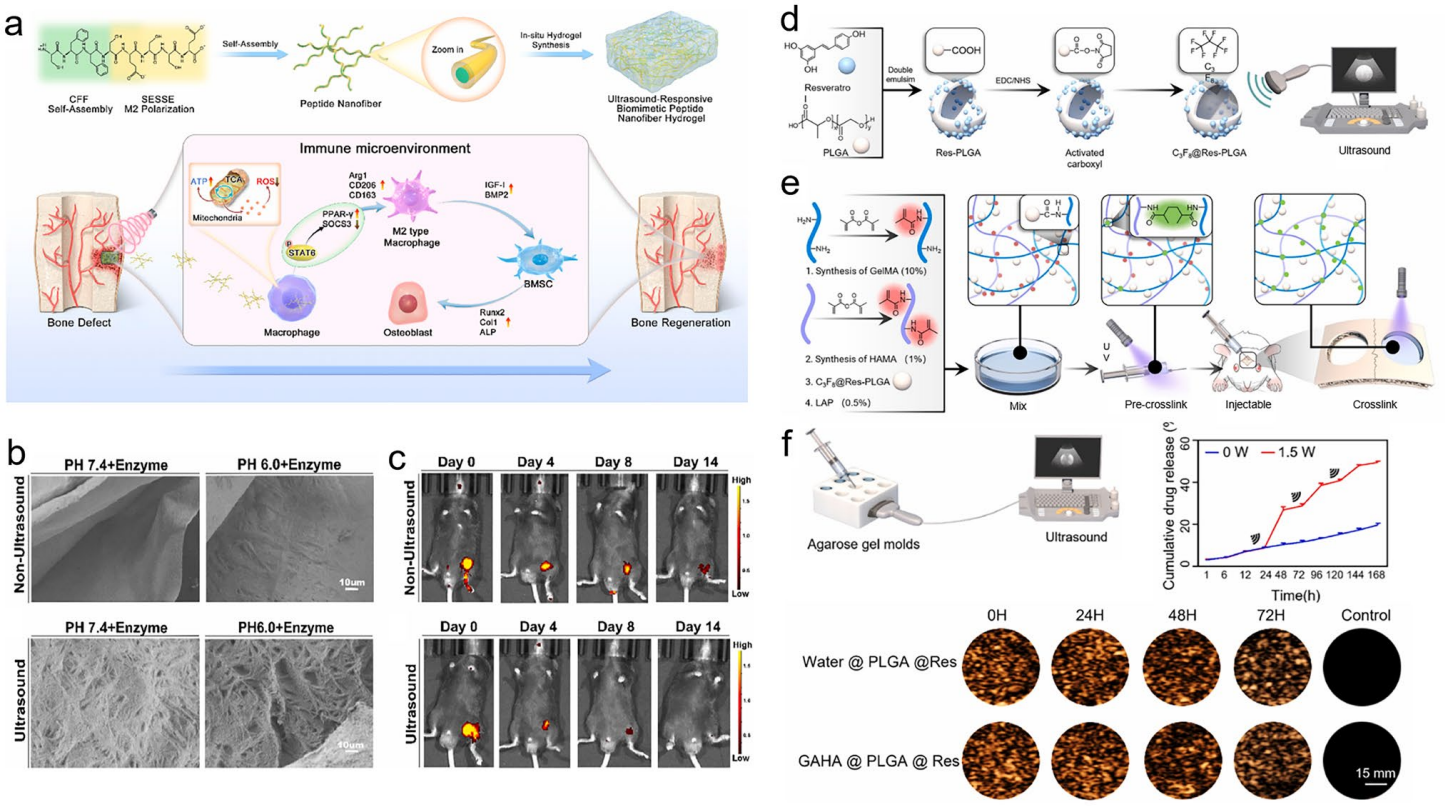
**a** Schematic illustration of the construction of a peptide nanofiber-loaded hydrogel and the mechanism of promoting osteogenesis using the hydrogels under US conditions. **b** SEM images of the peptide nanofiber-loaded hydrogel in different microenvironments with the presence and absence of US. **c** Use of US to accelerate hydrogel decomposition in vivo. **a**–**c** Reproduced under terms of the CC-BY license. Copyright 2023, Elsevier [32]. Illustrations of **d** the Res-decorated nanobubbles, and **e** fabrication of the nanobubble-incorporated hydrogels for cranial defect repair. **f** Diagram outlining the experimental design of the US process, and the Res release profile from the composite hydrogel. **d**–**f** Reproduced with permission. Copyright 2023, Elsevier [89]

US waves promote micelle disruption and drug release, mainly through the generation of shear stress [247]. In addition, US has been widely applied to accelerate bone regeneration because of its favorable tissue penetration ability [248]. Recently, the development of US-controlled drug delivery systems has achieved significant research attention. For example, Zhang et al. synthesized an US-responsive ultrashort peptide nanofiber (UPN)-incorporated alginate hydrogel (UPN@hydrogel) by coordinating the carboxyl groups of SA with Ca^2+^ (Fig. 13a). Under US stimuli, the UPN@hydrogel decomposed gradually with coordination disruption and released UPN from the hydrogel system (Fig. 13b, c). Consequently, M2 polarization was promoted to secrete BMP-2 and IGF-1, thereby accelerating osteogenic differentiation of the BMSCs, and achieving bone regeneration [32]. Using microfluidic techniques, Chen et al. prepared a US-responsive GelMA/heparin methacrylate (GelMA/HepMA) hydrogel microsphere by embedding oxygen-loaded nanobubbles, and the GelMA/HepMA microspheres were further functionalized with BMP-2 via non-covalent binding. Upon increasing the US intensity (i.e., 1, 2, 3, and 4 W), the released oxygen concentrations increased to 1.63-, 1.95-, 2.11-, and 2.29-fold, respectively. As a result, this microsphere system could precisely regulate oxygen release under US stimuli in vitro for a period of 9 h, maintaining a high VEGF level around the bone defects to provide nutrients for bone repair, while simultaneously delivering BMP-2 to boost femoral defect healing [33]. In addition, Han et al. [89] developed Res@PLGA nanobubbles-loaded Gel-HA methacrylate hydrogels (Fig. 13d, e), which released Res from the nanobubbles under US stimuli at 1.5 W cm^−2^. The rate of Res released reached 38.14%, and the nanobubbles presented a stable US-responsive capability in both water and in the hydrogel (Fig. 13f). Furthermore, Yi et al. [249] developed a hybrid hydrogel of SA containing sucrose acetate isobutyrate and naringin-loaded PLGA microspheres through Ca^2+^ crosslinking and realized on-demand delivery under US stimuli to accelerate cranial defects repair. These studies highlight the practicality of US stimulation for the intelligent and spatiotemporal delivery of drugs and bioactive molecules to promote bone regeneration.

##### Inflammation-Responsive Delivery

**Fig. 14:**
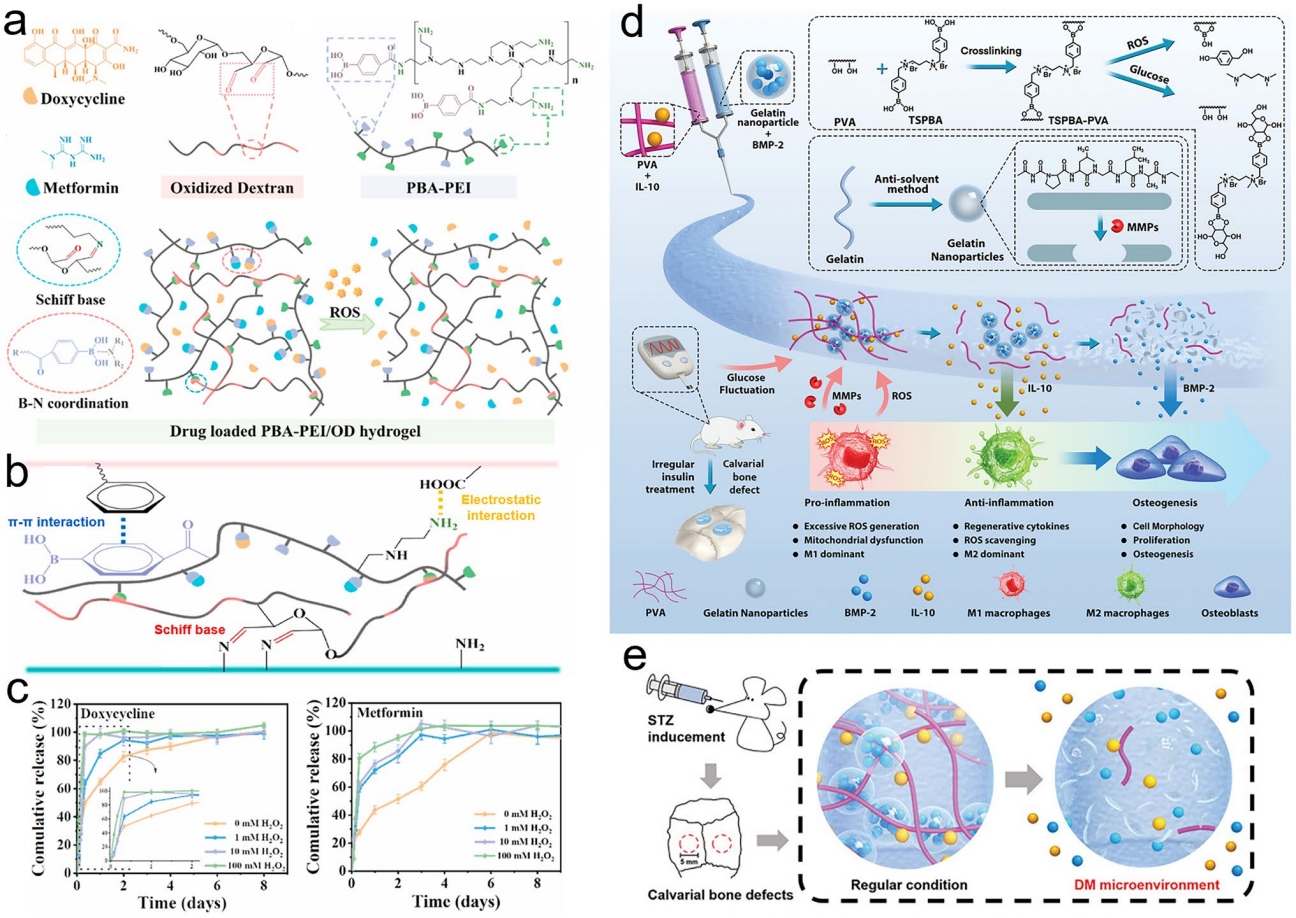
**a** Schematic illustration of the preparation of the Doxy- and Met-loaded PBA-PEI/OD hydrogel and ROS-triggered hydrogel decomposition. **b** Adhesion mechanism between the tissue and the hydrogel. **c** Cumulative release curves of Doxy and Met. **a**–**c** Reproduced with permission. Copyright 2022, Elsevier [90]. **d** Fabrication of the BMP-2 and IL-10-loaded ROS-responsive hydrogel. **e** Schematic diagram of BMP-2 and IL-10 released from the hydrogel under a diabetic microenvironment. **d**, **e** Reproduced with permission. Copyright 2022, John Wiley and Sons [253]

The inflammatory microenvironment around bone defects is usually acidic in nature and tends to attract many macrophages [250]. In addition to triggering severe inflammatory reactions at the bone defects, macrophages also play an important role in promoting osteogenesis [251]. ROS-responsive drug delivery systems capitalize on the distinctive redox microenvironment of tumors or inflammation, showing promise in biomedical applications, such as in targeted drug delivery for cancer and cell therapy platforms to address inflammation-related diseases. This section focuses on the current research and applications of inflammation-responsive hydrogel delivery systems for bone regeneration. In one study, Wang et al. developed a proanthocyanidin (PC)-coordinated ZnO microsphere composite thioether-grafted SA (TSA) hydrogel (ipPZCH) via CaCl_2_ crosslinking for use in the repair of infected bone tissue. In a high ROS microenvironment, the hydrophilicity of TSA was improved, inducing decomposition of the ipPZCH network to release PC-coordinated ZnO, and confer the hydrogel with an antimicrobial capability. In addition, the PC removed the overproduced ROS, while Zn^2+^ played a vital role in the osteoinductive process [30]. Moreover, Sun et al. designed ROS-scavenging and responsive oxygen-generating GelMA hydrogels incorporated with the antioxidant catalase (CAT) enzyme and oxygen-delivering NPs co-loaded liposomes (CPP-L). Under hypoxic conditions, the CPP-L/GelMA hydrogel released CAT to degrade H_2_O_2_ and generate O_2_. Subsequently, it sustained the delivery of oxygen for over two weeks in response to excessive ROS levels, thereby ensuring enhanced angiogenesis and osteogenesis [31]. Furthermore, Liu et al. fabricated a self-assembled nanomicelle by sequentially conjugating a polyethylene glycol chain and five phenylboronic acid pinacol esters to each hexachlorocyclotriphosphazene scaffold (denoted as PP5). The nanomicelles underwent ROS-triggered hydrolysis to eliminate the ROS, and subsequently, PP5 and rhBMP9 were incorporated into a poloxamer 407 (PX) hydrogel for mandibular defects repair [27]. The B-N coordination method has also been shown to enhance the drug-loading rates of hydrogels, thereby rendering it a common choice in conjunction with the ROS-responsive hydrogel delivery system. For example, Zhao et al. [90] developed an injectable local drug delivery system (LDDS) consisting of oxidized dextran (OD) and phenylboronic acid-functionalized poly(ethylene imine) (PBA-PEI) to improve the loading efficiency of Doxy and Met through B-N coordination, finally achieving ROS-triggered drug release (Fig. 14a, c). The sustained release of Doxy and Met was demonstrated to relieve inflammatory reactions and accelerate periodontal bone repair in chronic periodontitis model. Later, matrix metalloproteinase (MMP)-responsive is reported as a novel delivery strategy for responding to inflammatory microenvironments, and it has garnered significant attention from researchers to date. More specifically, Xu et al. [252] prepared a hydrogel consisting of triglycerol monostearate/2,6-di-*tert*-butyl-4-methylphenol (TM/BHT) and copper tannic acid coordination NSs (CuTA NSs) to promote osteogenesis under periodontitis conditions. This hydrogel exhibited MMP-responsive properties, was degraded under the chronic inflammatory conditions of periodontitis, and facilitated the on-demand therapeutic release of the CuTA nanozyme to induce M2 polarization and upregulate the expression of osteogenesis genes, ultimately enhancing tissue regeneration in periodontitis. Additionally, Li et al. [253] proposed a reversible poly(vinyl alcohol) (PVA) network through incorporating a colloidal network assembled from Gel NPs for application in diabetic bone regeneration. This PVA network degraded upon exposure to ROS and high glucose concentrations to undergo MMP-triggered degradation. Initially, the hydrogel delivered IL-10 to promote immunomodulation, and subsequently released BMP-2 to activate the osteoblasts and facilitate osteogenesis (Fig. 14d, e). It is widely known that various bone defects experience high H_2_O_2_ microenvironments after bone injury. To address this problem, He et al. [26] integrated rapamycin (Rapa)-loaded poly(diselenide-carbonate) nanomicelles with PEGylated poly(glycerol sebacate) (PEGS-NH_2_)/poly(*γ*-glutamic acid) (*γ*-PGA) to prepare an injectable hydrogel for aged bone regeneration. The release rate of Rapa from the hydrogel improved in proportion to the H_2_O_2_ level. The microenvironments around bone tumors are usually weakly acidic but contain high H_2_O_2_ levels. Thus, using 3D printing combined with a layer-by-layer assembly technique, Jiang et al. [28] designed an acidic/H_2_O_2_-responsive Gel scaffold by introducing PDA-hybridized ZIF-8 (pZIF-8) and PDA-decorated HAp NPs (pHAp NPs) to enhance tumor removal and bone repair. The encapsulation of cisplatin and BMP-2 into pZIF-8 resulted in drug delivery that was triggered by the tumor microenvironment. More specifically, under weakly acidic conditions and high H_2_O_2_ concentrations, the scaffold released cisplatin and BMP-2 to inhibit tumor growth and accelerate osteogenic differentiation, further enabling new bone formation through the acid/H_2_O_2_-induced cleavage of pZIF-8. In this system, PDA conferred the framework with an oxidative responsiveness capability, while H_2_O_2_ broke down the PDA chains into pyrrole-2,3-dicarboxylic acid and pyrrole-2,3-decarboxylic acid, thereby creating an acidic microenvironment to enhance the degradation of pZIF-8 on the scaffold. Consequently, the gradual delivery of cisplatin and BMP-2 led to the inhibition of bone tumor recurrence and boosted bone defect healing.

##### Sequential Delivery

Wound healing can be roughly divided into three stages, namely anti-inflammation, vascularization, and tissue reconstruction. The in vivo conditions necessary for repairing damaged tissues generally require the presence of various bioactive molecules and biological molecules, each engaging sequentially at the appropriate time and in effective doses [114]. Various key bioactive factors are known to play important roles in the different stages of bone repair, and so the spatial release of different molecules may promote the bone healing stage. In particular, numerous strategies have been developed to achieve the sequential release of bioactive molecules during bone repair, including bone-mimicking hydrogels with multifunctional carriers or multilayer structures.

**Fig. 15:**
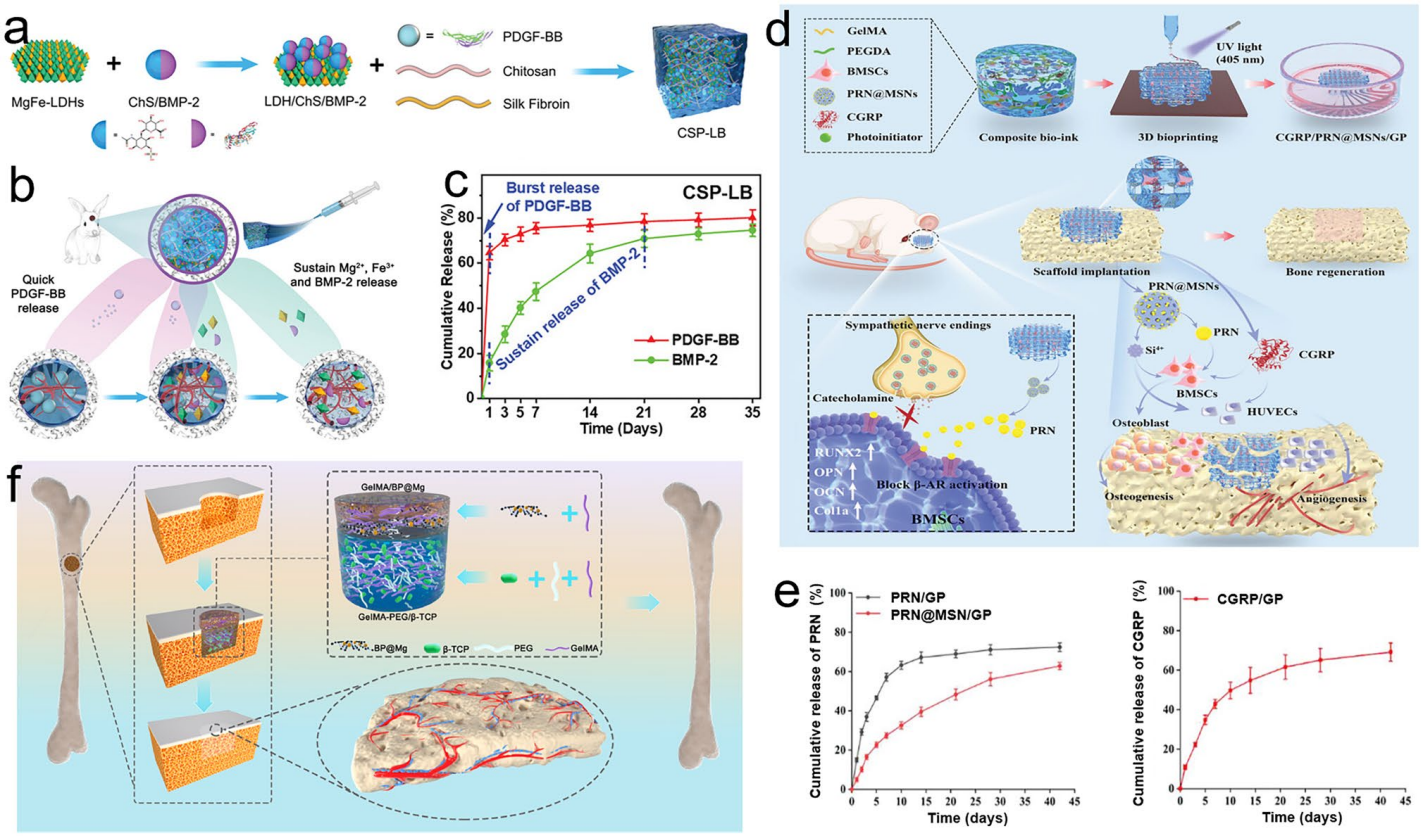
**a** Synthesis of the BMP-2- and PDGF-BB-encapsulated hydrogels based on MgFe-LDH NSs. **b** Schematic diagram and **c** release curves for the burst release of PDFG-BB and the sustained release of BMP-2 from the hydrogel to achieve spatial delivery at the lesion site. **a**–**c** Reproduced with permission. Copyright 2022, John Wiley and Sons [258]. **d** Preparation of PRN-laden MSNs and co-blended with the CGRP composite GelMA/PEGDA hydrogel to reconstruct the neuromodulatory microenvironment for cranial defect repair. **e** Release profiles of PRN and CGRP from the MSN/GP and GP hydrogels. **d**, **e** Reproduced with permission. Copyright 2023, John Wiley and Sons [85]. **f** Fabrication of a bilayer hydrogel composed of a GelMA-BP@Mg hydrogel layer (for periosteal repair) and a GelMA-PEG/*β*-TCP hydrogel (for bone repair) for reconstruction of the nerve-vascular network. Reproduced under terms of the CC-BY license. Copyright 2022, Elsevier [109]

In the context of carrier-assisted sequential delivery, multifunctional carriers include layer-by-layer assembled particles and core–shell structured particles. To comply with bone repair process, Tang et al. [53] fabricated a sandwich-like quaternary ammonium chitosan/Pluronic^®^F127 hydrogel containing abundant aldehyde groups via Schiff-base crosslinking. In this delivery system, VEGF was rapidly released from the hydrogel, and the sustained release of PDGF-BB from the outer surface of the CS microspheres with a low crosslinking density was achieved. Consequently, the long-term delivery of BMP-2 was achieved from the inner surfaces of the CS microspheres with high crosslinking densities. The results obtained for skull repair experiments revealed that the sequential release of three growth factors led to successful reconstruction of the cranial defect within 28 days. Similarly, Moncal et al. [254] reported a co-delivery system that achieved a burst release of platelet-derived growth factor-B encoded plasmid-DNA (pPDGF-B) from bioprinted constructs in 10 days, and which subsequently ensured the sustained release of pDNA encoded with BMP-2 (pBMP-2) from the CS NPs over 5-week period. Microspheres have also been applied in the context of sequential delivery. More specifically, Xu et al. [91] introduced Sema3A-encapsulated PLGA microspheres into a methylene blue (MB)-loaded dopamine-modified SA hydrogel (denoted as MB/Sema3A@SA-DA) and reported the sequential release MB initially (within 14 h) and Sema3A (within 28 days). Initially, the release of MB upon 660 nm laser exposure effectively suppressed bacterial growth and created an eligible microenvironment for new bone formation. Subsequently, the release of Sema3A promoted M2 polarization and osteogenesis. In another study, Lee et al. [255] introduced ADA-loaded microspheres and BMP-2 into Col-HAp composite scaffolds to fabricate a sequential dual-drug delivery system. In vitro experiments demonstrated that BMP-2 exhibited a rapid release behavior in the early stages, followed by the sequential release of ADA after 2 weeks. Owing to the synergistic effects of BMP-2 and ADA, effective bone regeneration was observed at 8 weeks after implantation in rats with 8-mm critical-sized cranial defects. Moreover, Nie et al. [256] incorporated VEGF-loaded PLGA microspheres and monocyte chemoattractant protein-1 (MCP-1) into a GO-reinforced poly(N-isopropylacrylamide)/CS (pNCG) hydrogel to fabricate a sequential release system of dual GFs for enhanced angiogenesis. Subsequently, vascular endothelial cells (VECs) were incorporated into the pNCG hydrogel matrix via sol–gel transition. In vitro and in vivo experiments demonstrated that the VECs could grow into the hydrogel network and simultaneously facilitate angiogenesis. In addition to microspheres, microparticles (MPs) have also been used to fabricate sequential release systems. For example, Park et al. [257] designed a dual-delivery system by sequentially releasing BMP-2 and IGF-1 from MPs that were incorporated into an injectable SA/Col-based hydrogel. BMP-2 was encapsulated into the Gel MPs to achieve quick release over 28 days, while IGF-1 was loaded into the PLGA-PEG-carboxyl (PLGA-PEG-COOH) MPs to achieve long-term delivery because of the different material degradation rates. After beam sterilization, the encapsulation efficiencies of BMP-2 and IGF-1 were both > 50% of the total GFs, and they remained active within the MPs. At a time of 4 weeks after hydrogel implantation, 8 mm cranial defects were successfully repaired to an extent, comparable to that achieved by the release of high-dose BMP-2 in a single dose; this result indicated that IGF-1 supplementation decreased the BMP-2 usage. And also, Lv et al. [258] combined burst and sustained delivery strategies by introducing BMP-2-modified MgFe-layered double hydroxide (LDH) NSs into a PDGF-BB-laden CS/SF hydrogel for cranial defect repair (Fig. 15a). The hydrogel realized the burst release (80.03%) of PDGF-BB from the hydrogel during the initial 7 days, in addition to the sustained release (74.51%) of BMP-2 from the NSs for 35 days, further inducing synergistic efficacy between angiogenesis and osteogenesis (Fig. 15b, c). Nanomaterials also can play a role in achieving burst release. For example, Li et al. [54] fabricated core–shell NPs (MDA NPs) consisting of MgO NPs as the core layer and 2-aminoethyl methacrylate-grafted PDA as the shell layer. These core–shell NPs were subsequently added to a phosphonate-functionalized methacrylamide CS/PAAm (CMP@PAAm) hydrogel network to develop a nanocomposite hydrogel for critical-sized cranial defect healing. Upon degradation of the core–shell nanoparticles embedded in hydrogel would be degraded to sustained release Mg^2+^ for 28 days at least. The Mg^2+^ release procedure was divided into two parts: burst and slow release. At the early stage, the MDA NPs rapidly decomposed in an aqueous solution to release large number of Mg^2+^, less Mg^2+^ still remained within the hydrogel network by the chelation interactions of phosphonate groups. Consequently, the chelated Mg^2+^ could be delivered slowly because of hydrogel degradation after 7 days, matching the proceeding of osteogenic activity in vivo. Furthermore, inspired by “flowerbed” characteristic, a nanofiber aggregates enhanced GFs delivery scaffold was developed based on 3D printing and electrospinning techniques. This scaffold was fabricated by introducing DMOG-laden mesoporous silica NPs and Sr^2+^-modified HAp into PLGA/Gel nanofibers (DMSN@PG) and PCL microfilaments (denoted as SrHAp@PCL), respectively, finally addition of DMSN@PG into SrHAp@PCL scaffold to gain flowerbed-like delivery system. Owing to different degradation rate of electrospun nanofibers and printed microfilaments, DMOG and Sr^2+^ could be released sequentially with the cleavage of scaffold [77]. In the same way, Guo et al. [85] co-blended PRN-loaded mesoporous silica NPs (MSNs) and CGRP into GelMA/PEGDA (GP) hydrogel scaffold. In this delivery system, PRN was released from the MSNs and next from the scaffold, which lasted over 42 days to enhance long-term bone regeneration. CGRP realized direct and rapid release from the scaffolds. Consequently, sequential delivery of PRN and CGRP could be contributed to accelerate angiogenesis and osteogenesis simultaneously (Fig. 15d, e).

In the context of assisted delivery using stratified structures, to mimic the in situ periodontal tissue location and achieve complete and concurrent regeneration at the lesion, Sowmya et al. [55] designed a nanocomposite hydrogel scaffold with a tri-layered structure, including a cementum layer (chitin-PLGA/nano-BG ceramic (nBGC)/cementum protein 1), a periodontal ligament (PDL) layer (chitin-PLGA/FGF-2), and an alveolar bone layer (chitin-PLGA/nBGC/PRP-derived GFs). This tri-layered scaffold permitted the spatial delivery of GFs to promote the formation of cementum, fibrous PDL, and alveolar bone tissue. Mimicked by the vascular nerve network, Xu et al. [109] established a bilayer hydrogel consisting of Mg^2+^-modified BP NSs incorporated into GelMA (upper layer) and a *β*-TCP nanocrystal containing GelMA/PEG hydrogel (bottom layer) for use in neuro-vascularized bone repair (Fig. 15f). They found that the Mg^2+^ released from the upper hydrogel layer enhanced angiogenesis, while the *β*-TCP nanocrystals released from the bottom hydrogel layer facilitated BMSCs differentiation toward osteogenesis. The demineralized bone matrix (DBM) generally consists of various osteogenesis-related bioactive molecules, mainly including BMP-2, COL I, and OPN, thereby leading to its application in bone injury treatment, including in a clinical setting. Considering that hydrophobic drugs are poorly compatible with hydrophilic hydrogels because of their aggregation and precipitation, Zhang et al. [259] hierarchically encapsulated SIM into the negative Lap layer (SL) by electrostatic interactions and ion exchange. Subsequently, they integrated DBM particles to fabricate a supramolecular hydrogel; these particles demonstrated a good water dispersion capability within the hydrogel network. Analysis by Fourier transform infrared spectroscopy showed that the SL presented characteristic bands corresponding to both SIM and Lap, suggesting that SIM was successfully loaded into the Lap layer. In addition, the slow-release behavior of SIM was confirmed, reaching 81.2% release in a neutral PBS solution on day 42. Importantly, it was found that this DBM-loaded hydrogel promoted new bone formation by upregulating BMP-2 expression. Furthermore, inspired by the structure of the osteon (the primary unit of cortical bone), which possesses self-organized spatiotemporal patterns due to reaction–diffusion dynamics, Wu et al. [260] fabricated an osteon-like hydrogel in a self-organized manner. In the cylindrical hydrogel, two opposite ion concentration gradients were formed in the peripheral and interior layers, and CaP achieved spatiotemporal self-organization in the concentric rings. The different layers possessed unique contents and crystalline phases, further guiding osteoblast adhesion and spreading in a manner similar to the function and behavior of osteons. Overall, this study provided a promising natural bone-like platform for bioactive molecule delivery.

Above all, hydrogel with high water content can load various bioactive molecules due to its porosity nature, then permits bioactive molecules release from hydrogel network via diffusion pathway, have been widely applied to proceed clinical trials of bone/cartilage defects healing. It can not only provide a simple strategy for increasing retention rate of bioactive molecules within bone defects, but also become a framework to support ingrowth of osteoblasts and chondroblasts within hydrogel matrix to replace bone defects with its gradual degradation. However, there are still having some disadvantages for various hydrogel system. (1) Conventional drug delivery methods remain widely used due to their simplicity, low cost, and ease of use, but their lack of specificity often leads to systemic side effects and suboptimal therapeutic outcomes. (2) On the other hand, nano-/microcarrier delivery systems offer significant advantages, such as enhanced targeting, improved bioavailability, and the ability to control drug release, thereby reducing side effects and improving treatment efficiency. However, these systems are often complex and expensive to manufacture and face regulatory and safety challenges. (3) Additionally, smart delivery systems represent the cutting edge of drug delivery, using advanced materials and technologies that respond to specific stimuli (like pH, temperature, or enzymes) to release drugs precisely at the target site. This approach maximizes therapeutic efficacy and minimizes off-target effects, but it also involves high development costs, complex design, and challenges related to scalability, stability, and regulatory approval. Overall, while nano-/microcarriers and smart delivery systems offer substantial improvements over conventional methods, their practical application requires overcoming significant technical, financial, and regulatory hurdles.

## Mechanical Properties of Hydrogel Delivery System

The mechanical properties of bone-related repair materials are crucial, given the role of bone as a load-bearing structure. The ideal bone repair material should possess mechanical strength that closely matches the natural bone’s Young’s modulus and compressive strength in the human body, typically around 15–20 GPa and 100–200 MPa for cortical bone, 0.1–2 GPa and 2–20 MPa for cancellous bone [261]. Hydrogels, which commonly mimic the structural properties of human soft tissue, face significant challenges in achieving the high mechanical strength required for bone repair. In clinical practice, bone filler materials typically have a relatively low elastic and compressive modulus, ranging from 0.01 to 3 GPa and 10–50 MPa, respectively [34]. Therefore, hydrogel delivery systems are more suitable as bone filler materials for clinical bone regeneration and repair.

Hydrogels can be categorized into four types based on their design: nanocomposite hydrogels, dual-network hydrogels, physically/chemically crosslinked hydrogels, and multi-designed hydrogels. For example, CaP nanocrystal was introduced into copolymerized hydrogel (poly(acrylonitrile-co-1-vinylimidazole), PAV) to develop an in vivo mineralized nanocomposite hydrogel, the mineralized PAV hydrogel showed tensile strength of 6.1 MPa, Young’s modulus of 6.47 MPa, compressive strength of 11.5 MPa, and fracture energy of 7935 J m^−2^, around twofold, 5.5-fold, 1.6-fold, and 2.0-fold that of the pure PAV hydrogel, respectively. The hydrogel achieved critical-sized cranial defects repair successfully. In this mineralized hydrogel, the synergistic effects of chemical crosslink of crosslinkers, CN–CN dipole–dipole interactions (for energy dissipation), and interfacial interactions between CaP nanocrystal and hydrogel matrix contribute to significantly improve the mechanical properties of PAV hydrogels [262]. In addition, Wu et al. [223] developed a nanocomposite hydrogel by incorporating trace amount of BMP-2-loaded PDA/heparin NPs into Gel/PEGDA/2-(dimethylamino)ethyl methacrylate system for mandibular bone regeneration. The nanocomposite hydrogel showed high compressive strength (higher than 0.7 MPa), apparently meeting the clinical requirements mentioned above. Furthermore, a SA-PAAm double-network hydrogel was proposed via ionic crosslinking, the compressive elastic modulus of this double-network hydrogel could be adjusted by different kinds of bioactive ions (including Sr, Ca, and Zn ions) and in vivo mineralization within hydrogel system, approximately 17.28, 2.68, and 5.85 MPa, respectively [263]. The mechanical performance of implanted hydrogel is generally weaker than that of the native bone tissue, they not only provide a mechanical and biological framework for cell ingrowth and oriented differentiation to enhance new bone formation, but also experience gradual degradation to leave space for new bone formation, finally the regenerated bone tissue achieves mechanical adaption to the natural bone.

Hydrogels, which exhibit both fluid-like (viscous) and solid-like (elastic) properties, provide a dynamic and tunable environment that closely mimics the natural ECM of bone tissue. The viscoelastic properties of the hydrogel, such as stiffness, stress relaxation, and mechanical adaptability, directly affect cell adhesion, proliferation, migration, and differentiation, which are critical for effective bone regeneration. Previous research has shown that MSCs in hydrogels tend to differentiate into adipocytes at elastic moduli of 1–10 kPa, while differentiation into osteoblasts occurs at moduli of 11–30 kPa [264]. Moreover, faster stress relaxation in hydrogels was found to promote osteogenic differentiation, indicating that MSC fate is not solely determined by the integration of elastic modulus over time or by cell shape [265]. Additionally, the dynamic mechanical properties of viscoelastic hydrogels have proven to be an effective strategy for enhancing the therapeutic potential of MSC spheroids in bone formation and repair [266]. Therefore, we believe that the viscoelastic properties of hydrogel delivery systems have significant implications for their application in biomedical engineering for bone repair.

## Conclusions and Perspectives

Bone healing involves long and complex processes that incorporate multiple interactions between molecular and cellular signaling pathways. These pathways are regulated by various bioactive molecules, such as drugs, GFs, bioactive ions, and EVs. The accumulation of sufficient bioactive molecules at the lesion site can ensure that the bone regeneration process progresses favorably. However, owing to their high mobilities under physiological conditions, the direct administration of bioactive molecules to injured sites can lead to negative phenomena, such as a low bioavailability, and potential side effects. In recent decades, hydrogel-based delivery platforms have represented a promising frontier in the field of bone regeneration, owing to their unique properties and versatility. These delivery platforms exhibit a range of desirable characteristics, including the stability to prolong the bioactivities and effective times of bioactive molecules due to their tissue-mimicking structures and properties. In addition, hydrogel-based delivery systems enable the in situ sustained release of bioactive molecules at the wound site, increasing the efficiency of drug utilization and thereby reducing healthcare costs. Furthermore, they can achieve sequential and responsive release of bioactive molecules to address the requirements of all stages of bone healing.

However, despite the advantages of hydrogel-based delivery platforms, significant challenges remain, and further investigation are required prior to their application in clinical treatments. For example, hydrogel-based delivery platforms have been criticized for their poor mechanical strengths, particularly in applications involving bone support, whereby achieving the strength of the original bone is challenging. In addition, despite the importance of the shape adaptability of the hydrogel delivery platform at the bone defect site is also crucial for its clinical application. However, there is a notable lack of research in this area. Indeed, maintaining good mechanical properties and an acceptable adaptability remain significant and ongoing challenges. To maintain good mechanical properties of hydrogels for bone regeneration, several strategies can be employed, including optimizing crosslinking density (both chemical and physical) to enhance strength and stability, incorporating reinforcing agents like nanomaterials or fibers to improve compressive and tensile strength, and designing composite hydrogels or interpenetrating polymer networks that combine the mechanical advantages of multiple polymers. Additionally, creating hydrogels with mechanical gradients or layered structures can better mimic the natural transition from soft to hard tissue, while controlling degradation rates ensures the scaffold maintains its integrity during tissue regeneration. Advanced methods such as 3D bioprinting to create defined architectures and developing self-healing hydrogels further enhance mechanical durability and resilience under physiological conditions. Moreover, the release of reconstruction-associated factors that promote vascular or neurological functions during bone repair has rarely been reported, and the strategic integration of multiple bioactive molecules within hydrogel-based delivery platforms is essential needed to advance the development of targeted and efficient therapeutic applications. Furthermore, the uncertain diffusion routes and contents of bioactive molecules in vivo increase the risk of adverse effects. Hydrogel-based delivery platforms should therefore be meticulously engineered to meet the physiological and therapeutic requirements of the individual patient, such as through the development of distinct delivery systems and tailored bioactive molecule formulations to effectively treat diseased and traumatic bone defects. Continuous efforts are therefore required to improve the clinical translation of the various hydrogel-based delivery platforms described herein.

Nowadays, a large number of hydrogel-based repair materials have entered clinical trials and clinical applications in recent years. For example, JointRep^®^ (NCT04840147) from Oligo Medic company is current undergoing clinical trials for the repair of cartilage defects from 2021 to 2025s. JointRep^®^ is fabricated on the basis of natural polymer CS and consists of an injectable thermo-gelling aqueous composition that enhances solid and sticky CS hydrogel formation when applied to the cartilage defects. Additionally, loxoprofen sodium hydrogel patch (LX-P, NCT03800797) has been used to test its therapeutics on ankylosing spondylitis treatment; the clinical trials have proceeded to the Phase 4. Moreover, mimicked by the natural bone matrix, a kind of collagen ceramic osteoconductive scaffold (Integra Mozaik™) has been developed and put into clinical applications, consists of 80% *β*-TCP and 20% COL I. The *β*-TCP and collagen I endow the scaffold with bone filler space and osteoconductive function, respectively. Although many hydrogel system products are already in clinical use, they still have certain limitations, such as heat-sensitive curing processes that can severely damage the activity of bioactive molecules, and inadequate mechanical properties. Therefore, the research and development of new, biosafe hydrogel delivery systems remain a significant challenge.

In summary, hydrogels are promising candidates for serving as delivery platforms for bioactive molecules in bone regeneration. For this purpose, cutting-edge medical technologies, including machine learning, artificial intelligence-assisted research and development (AI-assisted R&D), and the integration of surgical robotics, should be harnessed to broaden the functionality and application scope of hydrogel delivery platforms. Indeed, machine learning has recently been developed to generate a structurally diverse hydrogel library with more than 2,000 peptides, and to evaluate their corresponding properties for biomedical applications [267]. Additionally, the design of future hydrogel delivery platforms should consider factors such as patient age and health, ensuring their safer and more effective application across diverse patient populations. Overall, long-term interdisciplinary collaborations should be leveraged to develop intelligent hydrogel-based delivery platforms aimed at reducing patient pain and enhancing their overall quality of life.
